# The Invisible Footprint of Climbing Shoes: High Exposure to Rubber Additives in Indoor Facilities

**DOI:** 10.1021/acsestair.5c00017

**Published:** 2025-04-24

**Authors:** Anya Sherman, Thibault Masset, Lukas Wimmer, Leah K. Maruschka, Lea Ann Dailey, Thorsten Hüffer, Florian Breider, Thilo Hofmann

**Affiliations:** † University of Vienna, Centre for Microbiology and Environmental Systems Science, Environmental Geosciences EDGE, 1090 Vienna, Austria; ‡ University of Vienna, Doctoral School in Microbiology and Environmental Science, 1090 Vienna, Austria; § EPFL - Ecole Polytechnique Fédérale de Lausanne, Central Environmental Laboratory, Institute of Environmental Engineering, ENAC, station 2, CH-1015 Lausanne, Switzerland; ∥ University of Vienna, Department of Pharmaceutical Sciences, 1090 Vienna, Austria; ⊥ University of Vienna, Doctoral School of Pharmaceutical, Nutritional and Sport Sciences, 1090 Vienna, Austria; # University of Vienna, Research Platform Plastics in the Environment and Society (PLENTY), 1090 Vienna, Austria

**Keywords:** rubber additives, climbing shoes, human exposure, air quality, 6PPD-quinone

## Abstract

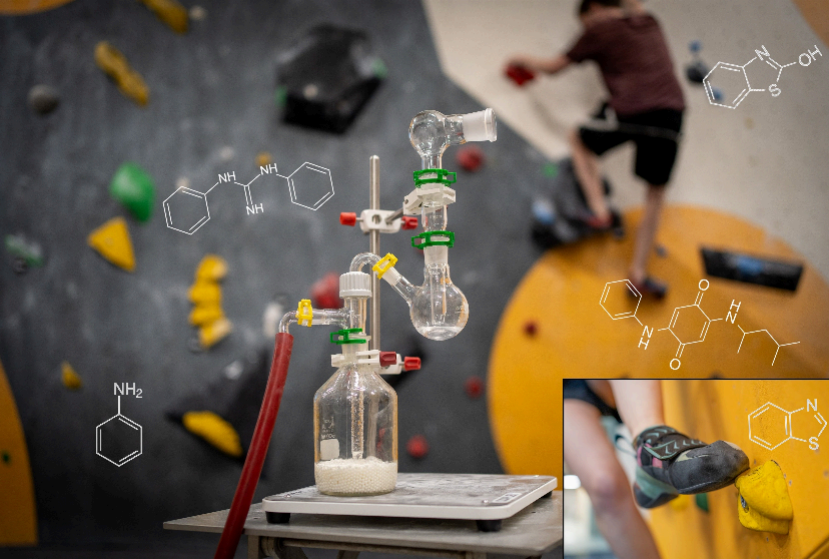

There is growing concern about rubber-derived compounds (RDCs), predominantly originating from tire and road wear particles. Other consumer products, including sports equipment, also contain RDCs, and human exposure to these compounds is of particular interest due to demonstrated toxicity to animal species. In this study, we investigated RDCs intentionally incorporated into climbing shoes for enhanced performance. We found high concentrations of 15 RDCs in shoe sole samples (Σ_15_ RDCs: 25–3405 μg/g), aerosol particulate matter (Σ_15_ RDCs: 2.6–37 μg/g), and settled dust (Σ_15_ RDCs: 1.5–55 μg/g) in indoor climbing halls. The estimated daily intake via inhalation/ingestion of Σ_15_ RDCs for climbers and employees in some of these facilities ranged from 1.7 to 48 ng/kg/day, exceeding known intake levels of RDCs from other sources. Abrasion powder resulting from friction between climbing shoes and footholds is the likeliest source of high concentrations of RDCs observed in aerosol particulate matter and settled dust. These findings reveal a previously unknown human exposure route of RDCs.

## Abstract 2

This work demonstrates how rubber abrasion from climbing shoes leads to the release of potentially toxic additives in indoor air.

## Introduction

**Figure 1:**
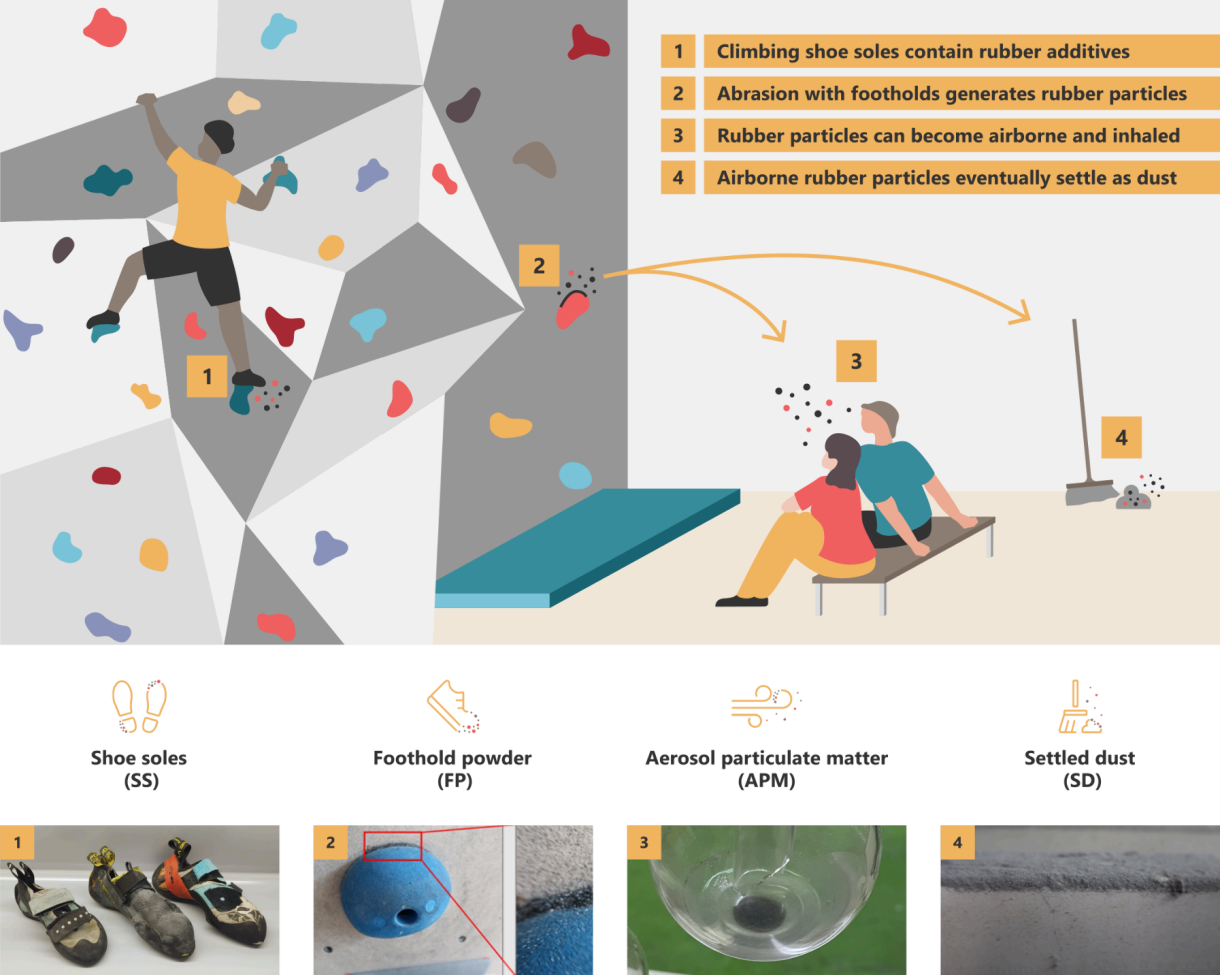
Schematic of a climbing hall, with photos of the four types of samples analyzed in our study. Specialized climbing shoes are worn with highly functionalized rubber soles (1, shoe soles). Friction between these shoe soles and the footholds generates rubber particles (2, foothold powder). Those can be aerosolized and inhaled directly upon generation, due to the brushing of holds, or by climbers falling onto mats and resuspending rubber particles that had settled (3, aerosol particulate matter). Eventually, aerosol particles also settle elsewhere as dust (4, settled dust).

Indoor air quality is a critical and increasing determinant of human health and is relevant not only in the home and workplace, but also in places of recreation, such as sports facilities.^1^ Indoor climbing is one increasingly popular form of indoor recreation. In indoor climbing halls, “handholds” and “footholds” are attached to specialized walls, allowing individuals to attempt to ascend the walls (Figure 1). In 2018, an estimated 1.5% of the UK population,^2^ and about 4.4% of the US population^3^ visited indoor climbing halls. Of these visitors, about 20% are regulars and spend several hours a day, multiple times a week in climbing halls.^2^

Several monitoring studies conducted in indoor climbing gyms reported very high particulate matter concentrations.^4,5^ Particulate matter exposure during indoor climbing is hypothesized to be the driver for acute decline in lung function of climbers.^5^ Chalk used by climbers is the primary source of particulate matter, but other sources may also contribute. In indoor halls, climbers wear specialized climbing shoes, with soles made of highly functionalized rubber. The rubber is chemically engineered to be flexible and sticky. Soles are intentionally designed to slowly abrade during climbing, due to desired friction with climbing holds. This leads to a constant generation of rubber particles, which accumulate on the climbing footholds. Most climbers own brushes to clean these climbing holds, which results in a constant aerosolization of rubber particles, which may remain airborne long enough to be inhaled by climbers or employees. The relative contribution of aerosolized rubber particles to total particulate matter remains uncertain but is likely minimal in comparison to chalk, which constitutes the primary source of airborne particles in indoor climbing facilities.^4^ Concerns regarding the potential health impacts of rubber particles may be more significant, as rubber typically contains a variety of chemical additives not present in chalk. This difference in chemical composition warrants further investigation into the specific risks posed by rubber particles in indoor climbing environments.

In tires, which are also highly engineered and abrade during their intended use, rubber-derived compounds (RDCs) concentrations are very high.^6^ Zhao et al. screened a wide range of elastomeric consumer products for multiple organic RDCs^7^ and several were found ubiquitously, although the concentrations in most consumer products were 1–2 orders of magnitude lower than in tires. The additive profile of elastomeric products was proposed to depend on the properties required for their intended use of the product.^21^ Due to the highly specialized properties of climbing shoes, we hypothesized that climbing shoes, and resulting abrasion particles, contain high additive concentrations. Indoor climbing halls may then be a hot spot of human exposure due to inhalation of airborne rubber particles. This is of concern, since RDCs such as 6PPD-quinone are also toxic to human lung cells^8−10^ with inhalation hypothesized to be a major route of exposure.

We collected aerosol particulate matter, settled dust and foothold powder samples in several climbing halls across Europe and measured the concentrations of RDCs therein. Based on measured aerosol particulate matter concentrations, we calculated the yearly exposure for employees and recreational visitors to these rubber-derived compounds. Our data indicate that climbing shoes are the source of these rubber-derived compounds.

## Materials and Methods

### Sample Collection and Characterization

Four types of samples were collected: aerosol particulate matter (aerosol PM), settled dust, shoe soles, and foothold powder (rubber powder accumulated on climbing footholds which results from the abrasion of climbing shoes). Complete sample sets (aerosol PM, triplicate settled dust, and triplicate foothold powder samples) were collected in five halls in Vienna. These five halls vary in size, age, number of visitors, and ventilation (Table S1). To assess the levels of RDCs more broadly, additional samples (triplicate settled dust and triplicate foothold powder samples) were collected in four more halls, in Switzerland, France, and Spain. In addition, 30 shoe sole samples representing major brands and models were analyzed. All samples were collected between February 2023 and June 2024.

Aerosol PM was collected with a standardized glass liquid impinger (Copley Scientific Ltd.) which is an active sampling device that separates aerosol PM into aerosols which predominantly deposit in the upper respiratory tract (>6.4 μm aerodynamic diameter; URT) and the fraction that deposits predominantly in the lower respiratory tract (<6.4 μm aerodynamic diameter; LRT). Liquid impingers have been shown to have a collection efficiency of up to 99% for particles in the 0.02–4 μm size range,^11^ and are explicitly mentioned by the World Health Organization as an acceptable device for sampling nano- and microplastics.^12^ However, collection efficiency of rubber particles was not validated within the scope of this study. Pooled aerosol PM samples (i.e., material collected from 3 h sampling runs) were collected in Hall 01 and Hall 02 on five consecutive days in April 2023 during peak activity (5 to 8 p.m.). Pooled aerosol PM samples were collected in Hall 03, 04, and 05 on five consecutive days in April and May 2024 during peak activity (5 to 8 p.m.). The air inlet was set at a height of 142 cm, facing the climbing wall at approximately 3 m distance. The air flow rate was 60 ± 2 L/min, for a total volume of 54 m^3^ air per sample in each climbing hall. Further details about aerosol PM sampling are provided in Supplementary Text S1.

Settled dust samples were collected from uncleaned floor and wood surfaces 5 to 10 m from the climbing walls. In each hall, settled dust from three distinct locations was sampled to assess spatial variability within the hall. Dust was collected using a clean metallic spatula and immediately placed in cleaned glass vials, then stored at −20 °C until further processing.

Thirty shoe sole samples were collected from both used and new climbing shoes (specialized rubber shoes worn while climbing, Figure 1) to represent the marketplace. Approximately 1 g of rubber was cut out from the tip of the sole using a ceramic knife. Samples were cut into 1 mm^3^ pieces and ground into fine powder using cryo-ball milling (MM400, Retsch) for 2 min at 25 Hz. After grinding, 50 mg powder was immediately suspended in 1 mL dichloromethane to prevent reagglomeration, and then extracted. Clean climbing hold and mat samples were obtained from Hall 02 and extracted via the same procedure as the shoe sole samples.

A cleaned metal spatula was used to collect 1 – 5 g of rubber powder accumulated in the clefts of climbing footholds (foothold powder samples). These foothold powder samples were immediately placed in cleaned glass vials and stored at −20 °C until further processing.

Subsamples of pure solid chalk, foothold powder samples and settled dust samples were coated with a gold nanolayer (10 nm) and visually characterized with a scanning electron microscope (Gemini SEM 300, Zeiss) at various magnification levels.

### Sample Extraction and Measurement

Liquid was removed from the aerosol PM samples via rotary evaporation (ethanol) and lyophilization (Milli-Q-water). The residual particle mass was determined gravimetrically using a high precision balance and samples were resuspended in ethanol. All samples were extracted with accelerated solvent extraction (Supplementary Text S2). The selection of RDCs for analysis was based on several criteria such as common use in the rubber industry, diversity in terms of chemical classes, availability of commercial standards for quantitation and potential toxicity for humans. Therefore, the following RDCs were analyzed in all samples with UPLC-MS/MS: benzothiazole (BTZ), 2-hydroxybenzothiazole (2OH-BTZ), 2-aminobenzothiazole (2-amino-BTZ), 2-mercaptobenzothiazole (2SH-BTZ), aniline, 1,3-diphenylguanidine (DPG), hexa(methoxymethyl)melamine (HMMM), and the phenylenediamine compounds: 6PPD, IPPD, CPPD, DPPD and their associated quinones: 6PPDq, IPPDq, CPPDq, DPPDq. Details are provided in the SI regarding the chemicals and internal standards used (Supplementary Text S3), UPLC-MS/MS instruments and methods (Supplementary Text S4).

### QA/QC

Blanks were collected at different stages of the sample processing workflow, to assess contamination that may have arisen during aerosol PM sampling (collection blanks), sample storage (storage blanks), and laboratory processing (laboratory blanks). QA/QC including extraction recovery, blank collection and processing are detailed in Supplementary Text S5 and Table S2.

To investigate background levels of RDCs, reference samples were collected in an administrative office of climbing Hall 02, which was in the same building, but not connected to the climbing area. Sampling procedure was the same as for the air sampling in the climbing areas. Finally, to account for other potential sources of RDCs in climbing areas, samples of climbing holds (grips used for feet and hands during climbing, typically made out of polyurethane or polyester), as well as two types of climbing mats from climbing Hall 02 were obtained and extracted (Supplementary Text S5).

### Exposure Calculations

To determine the human exposure to RDCs in climbing halls, estimated daily intake values via inhalation (and ingestion) were calculated using eq 1 for two types of individuals: regular adult climbers and employees working at the halls:
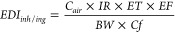
1where by *EDI*_*inh*__/*ing*_ is the estimated daily intake via inhalation (and ingestion) (ng/kg/day), *C*_*air*_ the concentration of RDCs in the total aerosol PM (ng/m^3^), *IR* the inhalation rate (m^3^/hour), *ET* the exposure time (hours/day), *EF* the exposure frequency (days/year), *BW* the body weight (kg) and *Cf* the number of days per year. Exposure parameters are available in Table S3, and were obtained from the US EPA exposure factor handbook.^13^ This calculation assumed that the RDCs in the LRT fraction represent inhalation exposure, while the concentrations measured in the URT fraction represent a combination of inhalation/ingestion exposure, since a high fraction of aerosol PM depositing in the extrathoracic/tracheal region of the URT is quickly cleared to the gastrointestinal tract.^14^

### Transformation Experiments

Ozonation experiments were performed to investigate potential transformations of RDCs in foothold powder. Foothold powder was collected from climbing Hall 01 immediately before experiment start and divided into six subsamples. An ozone chamber^15^ was employed to expose three subsamples to elevated ozone concentrations (1 g/m^3^) at room temperature for 4 h in the dark. Ozone concentrations were substantially higher than realistic indoor concentrations, which is typical for ozonation experiments.^16,17^ NO_X_ concentration was also measured to be 9 ppm during the experiment. After ozonation, all sub samples were spiked with internal standards, and extracted and measured with UPLC-MS/MS as described above.

## Results and Discussion

### Air and Dust Concentrations of Rubber-Derived Compounds and Associated Human Exposure in Climbing Halls

Air measurements carried out during peak activity hours revealed very high aerosol PM concentrations (Table 1). The concentrations of aerosol PM (from four climbing halls) ranged from 590 μg/m^3^ to 1,990 μg/m^3^ in the URT fraction (>6.4 μm) and from 890 μg/m^3^ to 1080 μg/m^3^ in the LRT fraction (<6.4 μm) (Table 1). Halls 01 and 03 had the highest aerosol PM concentrations, which is likely related to more climbing activity (more check-ins per hour) than Halls 02, 04, and 05. Hall 05 had by far the lowest measured aerosol PM concentrations, and the lowest number of check-ins per hour. The absence of ventilation in Hall 02 may explain its higher aerosol PM concentrations compared to Hall 04, despite similar sizes and check-ins per hour (Table S1). A relationship between activity and aerosol PM concentrations has been previously observed in climbing halls^4^ and gymnasiums.^18^ The aerosol concentrations reported in this study are in the same range as aerosol PM concentrations previously reported for indoor climbing halls (from 509 to 4,028 μg/m^3^, measured using an impactor with an aerodynamic size cutoff of 10 μm; PM_10_).^4^ These values exceed those of most other indoor environments^19^ including indoor artificial turf halls (31 to 40 μg/m^3^)^20^ and other sports environments.^1^ They are comparable to maximum reported PM_10_ concentrations in gymnastic facilities where chalk is also used (500 to 900 μg/m^3^).^21^

**Table 1:** Concentrations of Aerosol PM Measured in Halls 01–05

	hall 01	hall 02	hall 03	hall 04	hall 05
LRT fraction (μg/m^3^)	1040	900	1080	890	100
URT fraction (μg/m^3^)	1590	1000	1990	590	80
total aerosol PM (μg/m^3^)	2630	1900	3070	1480	180

Aerosol PM concentrations in this study are near current limits for occupational exposure to low-toxicity dusts (<4–10 mg/m^3^ inhalable fraction; < 1.5–4 mg/m^3^ respirable fraction over an 8-h time-weighted average exposure; limits are different for different countries).^22^

It is important to note that the presence of leachable RDCs in climbing hall dusts may pose a higher health risk than conventional low-toxicity dusts, thereby providing a compelling rationale for further investigations. Ten RDCs were detected above the limit of quantification in aerosol PM samples from Halls 01–04 (Table S4). Due to low particulate matter collected, RDC concentrations in aerosol PM from Hall 05 are not reported. A consistent set of RDCs dominated the chemical profile in all aerosol PM samples. Among these, four RDCs were identified as transformation products (TPs) of compounds commonly used in rubber products: 2OH-BTZ, BTZ, as well 6PPD-quinone were detected in the aerosol PM of all climbing halls, and aniline was detected in the aerosol PM of Halls 01 and 02. All transformation products were quantified using authentic standards. Cumulative RDC concentrations in the URT fraction ranged from 0.92 to 28.4 ng/m^3^, while RDC concentrations in the LRT fraction ranged from 1.10 to 7.81 ng/m^3^. Higher RDC concentrations were found in the URT fraction of aerosol PM, although in Hall 03, total RDC concentrations, as well as individual concentrations of 2OH-BTZ, BTZ, HMMM, and 6PPD were notably higher in the LRT fraction than the URT fraction (Table S4). It is important to mention that these mass-based concentrations are normalized to total APM mass, so the higher RDC concentrations in the LRT fraction in hall 03 could be due to a reduction in other sources of fine particulate matter in that hall rather than different characteristics of respirable rubber particles. In fact, RDC profile was quite similar between the URT and LRT fractions of all halls except in Hall 04 where benzothiazole was not detected in the LRT fraction (Figure S2).

**Figure 2:**
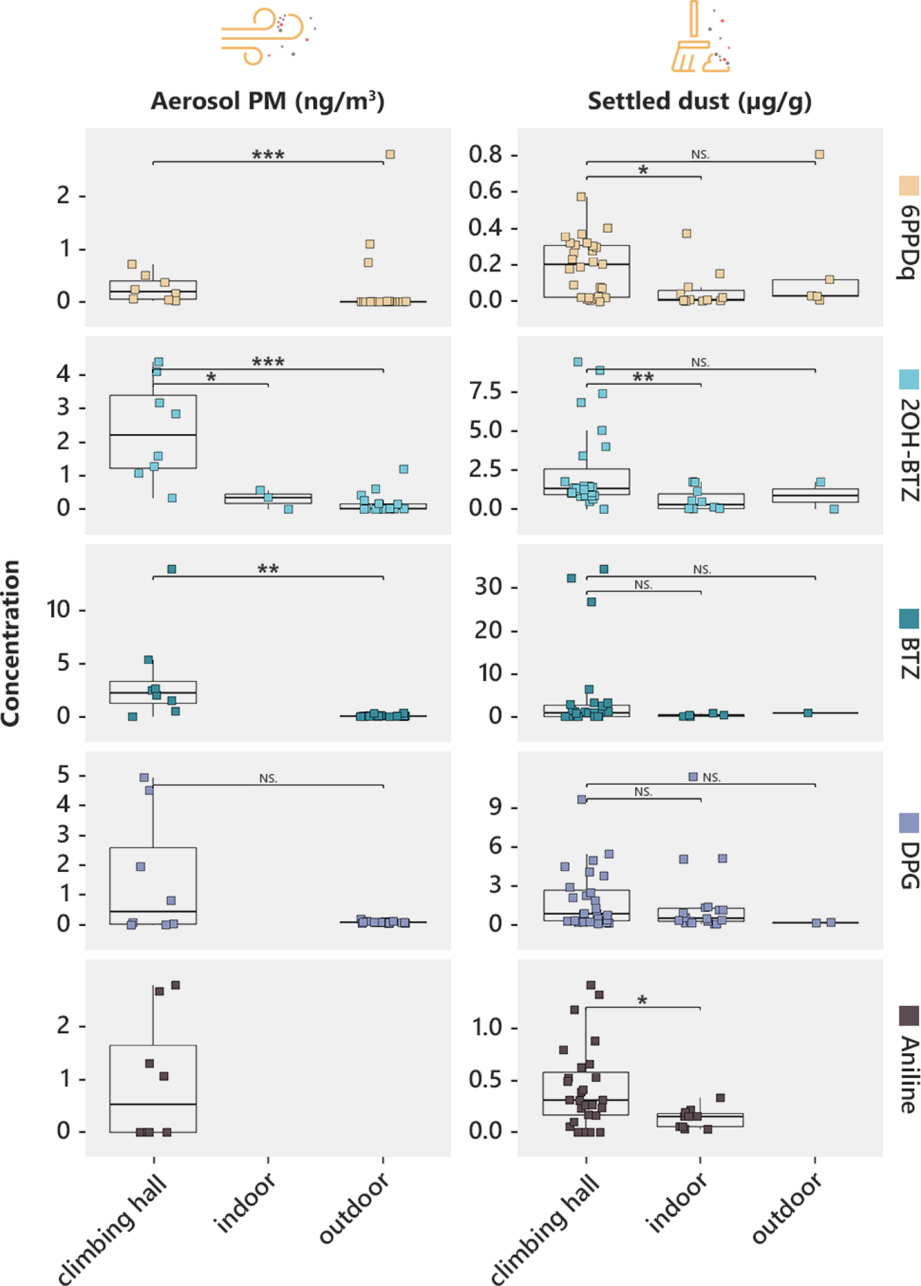
Comparison of rubber-derived compound concentrations in climbing halls and other environments.Rubber-derived compound concentrations measured in the LRT and URT fractions of climbing hall aerosol particulate matter (left) and settled dust (right) compared to concentrations reported in the literature for various indoor (houses, vehicles, shopping malls, dormitories, parking lot, and sport halls) and outdoor (roadsides, city centers, playgrounds, recycling plants, and industrial sites) environments. Aerosol particulate matter and settled dust concentrations compiled from the literature represent a variety of size fractions. Details about literature values, including size fractions of particulate matter and settled dust, are provided in the Supporting Information as an Excel file. Statistical differences between groups were tested with the Wilcoxon signed-rank test (NS means *p* ≥ 0.05; * means *p* < 0.05; ** means *p* < 0.01; *** means *p* < 0.001).

**Figure 3:**
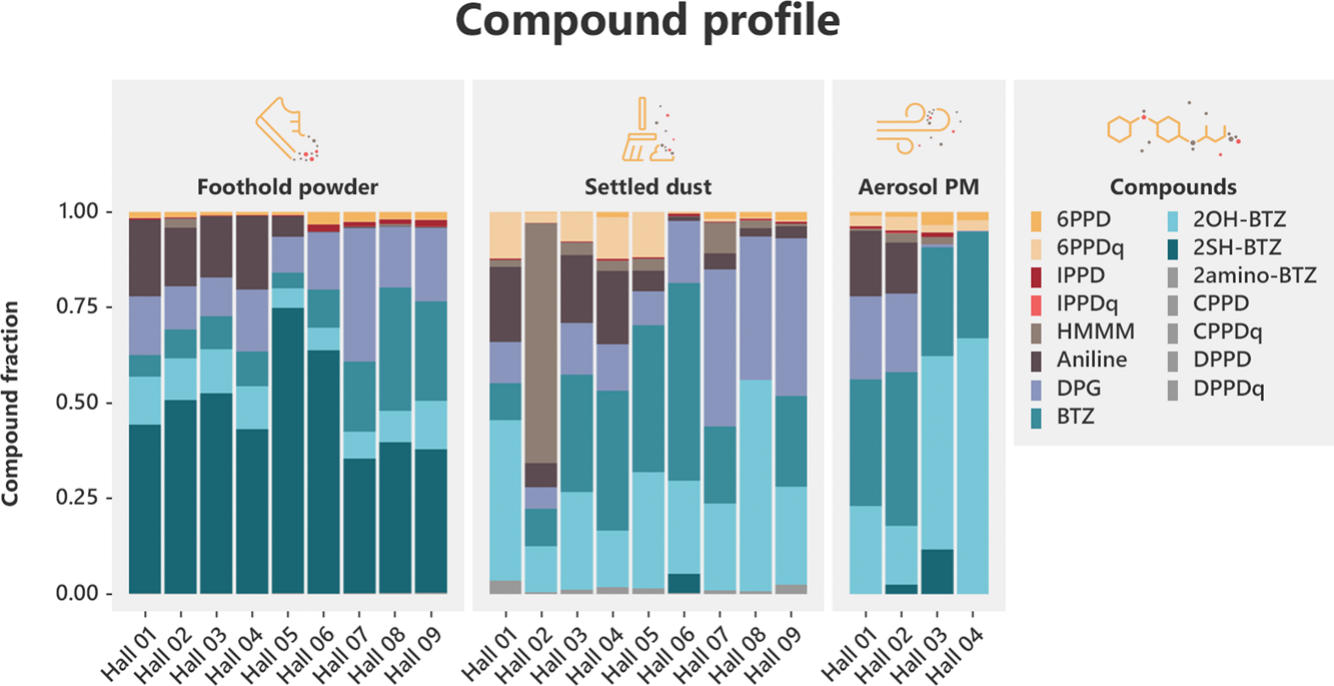
Rubber-derived compound profile shift. Rubber-derived compound profile in foothold powder, settled dust, and aerosol PM. Foothold powder and settled dust compound profiles for each hall represent an average of triplicate samples, and aerosol PM represents an average of the URT and LRT fractions. Individual, absolute concentrations for each sample are presented in Table S4. Aerosol PM was only collected from halls 01–05, and in hall 05, the collected mass of aerosol PM was too low for accurate analysis of RDCs.

It has been shown that concentrations in settled dust of organic compounds with similar physico-characteristics as RDCs (phthalate esters) tend to correlate with their concentrations in aerosol PM,^23^ thus, we quantified RDCs in dust samples from all climbing halls. We detected 14 out of the 15 RDCs (except CPPD-Q) in at least one triplicate sample from nine climbing halls investigated (Table 2). Since settled dust samples collected from different areas of a hall contain different amounts of rubber particles, substantial variability in total RDC concentrations was observed between triplicate settled dust samples (Table S4). However, the chemical profiles within dust samples from nine halls were consistent except in Hall 02 and were consistently dominated by the same chemicals, which dominated the profile of aerosol PM samples (Figure 3). Overall, cumulative RDC concentrations in settled dust samples were high (1.6 to 55 μg/g). The similarity in chemical profile of settled dust and aerosol PM samples from nine halls in four different countries (France, Switzerland, Spain and Austria) suggests an important source of RDCs which may be ubiquitously present in climbing halls. One exception to this similarity was the elevated concentrations of HMMM present in all sample types (particularly in settled dust) from Hall 02, suggesting an alternate source of HMMM in this hall. Hall 02 was built inside an old building, within the historical city center of Vienna, while all other climbing halls are constructed in newer buildings. Aside from its use in rubber, HMMM is used in production of plastics and metal coatings,^24^ and it is possible that building materials may contribute to the elevated concentrations of HMMM in Hall 02. It is worth noting here that reference samples of APM from offices in Hall 02 did not contain detectable HMMM concentrations.

**Table 2:** Rubber-Derived Compound Concentrations Measured in Bouldering Halls^a^

	LRT fraction (ng/m^3^)	URT fraction (ng/m^3^)	total aerosol PM (ng/m^3^)	settled dust (μg/g)	foothold powder (μg/g)
aniline	<LOQ–1.31	<LOQ–2.80	<LOQ–4.12	<LOQ–1.42	<LOQ–61.6
DPG	0.005–1.96	<LOQ–4.97	0.005–6.50	0.058–9.65	11.7–118.0
2-OH-BTZ	1.07–4.41	0.33–4.11	1.41–7.26	<LOQ–9.50	<LOQ–43.0
IPPD	<LOQ–0.05	<LOQ–0.14	<LOQ–0.18	<LOQ–0.38	0.11–22.6
BTZ	<LOQ–2.47	0.51–13.9	0.51–16.4	<LOQ–34.4	<LOQ–99.7
2-amino-BTZ	<LOQ	<LOQ	<LOQ	<LOQ–0.51	0.15–1.10
2-SH-BTZ	<LOQ–0.55	<LOQ–1.41	<LOQ–1.71	<LOQ–4.37	43.5–553
HMMM	<LOQ–0.25	<LOQ–0.50	<LOQ–0.75	<LOQ–17.0	<LOQ–6.64
CPPD	<LOQ	<LOQ	<LOQ	<LOQ–0.008	<LOQ–0.17
6PPD	<LOQ–0.24	0.04–0.31	0.04–0.49	<LOQ–0.30	0.61–33.8
IPPDq	<LOQ–0.02	<LOQ–0.04	<LOQ–0.06	<LOQ–0.042	<LOQ–0.42
DPPDq	<LOQ	<LOQ	<LOQ	<LOQ–0.047	<LOQ–<LOQ
6PPDq	0.02–0.37	0.04–0.71	0.05–1.08	<LOQ–0.58	0.11–0.91
CPPDq	<LOQ	<LOQ	<LOQ	<LOQ	<LOQ
DPPD	<LOQ	<LOQ	<LOQ	<LOQ–0.001	<LOQ

a Concentrations measured in aerosol PM and settled dust samples from all bouldering halls are shown as a range (aerosol PM data from hall 05 excluded).

Concentrations of most RDCs in settled dust samples were higher in some halls than those reported from other indoor environments (Figure 2, Figure S1). In some samples (particularly in Halls 06 and 09), BTZ concentrations were one or 2 orders of magnitude higher in our samples than in house dust and even road dust.^25−27^ 6PPDq concentrations were also higher than in most house dust samples collected around the world^28−31^ and were similar to road dust samples.^32,33^ Concentrations of DPG were also high (up to 9.65 μg/g) and exceeded most reported DPG concentrations in house dust^34,35^ (Figure 2).

Similar to dust, the concentrations of most RDCs in the collected aerosol PM samples were very high, particularly in Halls 01, 02, and 03 compared to other atmospheric environments (Figure 2, Figure S1 and SI: Excel file containing concentrations of all RDCs). PPD and PPDq concentrations in the LRT fraction of this study were higher than those measured in the PM_2.5_ fraction of aerosols collected in Chinese megacities^36,37^ and similar to concentrations determined from PM_2.5_ fractions at roadside sites and city centers in China during air pollution events.^38^ Concentrations of DPG, BTZ, and 2OH-BTZ in aerosol PM samples collected in the LRT fraction were one or 2 orders of magnitude higher than those determined in the total particulate matter collected in 18 megacities worldwide^39^ and BTZ and 2OH-BTZ were up to 10-fold above concentrations measured in PM_10_ fraction of aerosols from industrial areas in Spain.^40^ So far, studies reporting RDC concentrations in aerosol PM collected in indoor environments remain very scarce. Dye et al. (2006)^20^ reported higher concentrations of 2amino-BTZ, 2SH-BTZ and IPPD but lower concentrations of 2OH-BTZ in PM_10_ collected in indoor artificial turf halls compared to our aerosol PM data. However, these data should be treated with caution, as they are not based on direct measurements but on RDC concentrations in ground granulate and estimated rubber concentrations in aerosol PM. This approach does not account for potential differences in chemical composition between airborne rubber particles and those on the ground, such as those resulting from atmospheric transformations. Additionally, the distinct rubber-derived chemical composition of turf granulate (often made from recycled tires) compared to climbing shoes may explain the discrepancies between the concentrations reported by Dye et al. and our measured concentrations.^41^

We calculated the estimated daily intake via inhalation and ingestion (EDI_inh/ing_) for adult climbers and employees working at the halls (Table 3; Table S3 for details). Mean EDI_inh/ing_ values showed that employees would be subjected to a higher exposure than climbers due to their longer average exposure time, despite their lower inhalation rate (Table 3 and Table S3). EDI_inh/ing_ for ∑benzothiazoles ranged from 0.04 to 29 ng/kg/day and exceeded EDI_inh/ing_ for ∑PPDs (up to 0.9 ng/kg/day) which were similar to EDI_inh/ing_ for ∑PPDqs (up to 1.5 ng/kg/day). Even though EDIs are presented as daily exposures, they account for exposure frequency throughout the year (see eq 1) making them directly comparable with EDI from the literature that evaluate chronic exposure to RDCs. The EDI_inh/ing_ derived for BTZs in this study was up to 2 orders of magnitude above those estimated for employees near industrial sites in Spain^40^ and exceeded dermal exposure through textile (EDI_dermal_ for ∑_3_BTZs = 244 – 395 pg/kg/day^42^). EDI_inh/ing_ for PPDs and PPDqs were up to 3.1 and 7.8-fold higher than EDI_inh_ for near-roadside workers in Chinese megacities and 2 orders of magnitude higher than the EDI_inh_ for the adult population in Hong-Kong.^37^ EDI_inh/ing_ for DPG ranged from 0.01 – 8.61 ng/kg/day exceeding in most cases EDI via household dust ingestion in 11 countries (0.0 – 0.9 ng/kg/day)^34^ (Table 3).

**Table 3:** Estimated Daily Intake via Inhalation/Ingestion of Rubber-Derived Compounds^a^

EDI (ng/kg/day)	aniline	BTZ	2OH-BTZ	DPG	HMMM	6PPD	IPPD	6PPDq	IPPDq
EDI_inh/ing_(employee)	nd–5.45	0.06–21.7	1.86–7.14	0.01–8.61	nd–0.99	nd–0.65	nd–0.24	0.07 −1.43	nd–0.08
EDI_inh/ing_ (climber)	nd–3.50	0.04–13.9	1.19–6.17	0.01–5.53	nd–0.63	nd–0.42	nd–0.16	0.05–0.92	nd–0.05
EDI by dust ingestion^34^ or roadside soil ingestion^37^				0.01–0.87^34^		Σ5PPDs		Σ5PPDqs	
						0.5 – 0.9^37^		0.70–1.10^37^	
EDI_inh_ ambient air (worker)		Σ5BTZs				0.19^43^	0.06^43^	0.13^43^	0.07^43^
		0.02–0.06^40^							
EDI_inh_ ambient air (adult residents)						Σ5PPDs		Σ5PPDqs	
						0.0002–0.0006^37^		0.0001 – 0.001^37^	

a Range of estimated daily intake (ng/kg/day) by inhalation from two subgroups (adult climbers and employees, 21–31 years of age) derived from halls 01–05 and based on aerosol PM data (combined LRT+URT fractions). nd, not determined due to aerosol PM concentrations < LOQ. Data are compared with EDI from multiple sources obtained from the literature.

It is important to note that the EDIs reported in this study are relevant for climbers who visit during peak hours. Aerosol PM concentrations in climbing halls vary greatly throughout the day, in correlation with the number of visitors present.^4^ Therefore, it can be expected that climbers visiting outside of peak hours will have lower exposure to RDCs via inhalation of aerosol PM.

### Engineered Climbing Shoes Contain Large Quantities of Rubber-Derived Compounds

Elevated RDC concentrations in the air of climbing halls and settled dust were not due to contamination, ambient RDC levels, or sources such as climbing holds or mats, as confirmed by the blank and reference sample data (Supplementary Text S5). We attribute the high concentrations of RDCs specifically to climbing activity. Laboratory blanks showed only trace amounts of 2SH-BTZ and 2OH-BTZ in one out of four samples. Collection and storage blanks contained low levels of 2SH-BTZ, 2OH-BTZ, BTZ, DPG, and IPPD (Section S5). This is expected, as the blanks were prepared in the climbing halls, where RDCs were present at high concentrations. Importantly, RDC levels in these blanks were at least an order of magnitude lower than those measured in aerosol PM samples, confirming that the majority of RDCs in our samples were not introduced via contamination.

In reference APM samples collected in offices of climbing Hall 02 (same building), where climbing is not practiced, we detected 6PPD and IPPD (0.10 and 0.86 ng/m^3^ respectively, Section S5) in the combined LRT + URT samples. This is consistent with expectations, as the concentrations of 6PPD and IPPD in these reference samples (offices) are in the range of those reported in other indoor^28^ and outdoor^35,47,50,53−56^ environments (Figure S1), implying that these RDCs are present at background levels, and not specifically derived from climbing activity. On the other hand, BTZ, 2OH-BTZ, 6PPDq, IPPDq, DPG, aniline and HMMM were not detected in the reference samples (Section S5).

Several potential climbing-related sources of RDCs were identified. IPPD was detected in one climbing hold (66.0 ng/g), and in one of the mats measured (83.1 ng/g). IPPDq was detected in the same mat (5.8 ng/g, Section S5). In contrast, dust samples contain < LOQ – 380 ng/g IPPD and < LOQ - 42 ng/g IPPDq. Climbing holds and mats are made from durable materials with very low abrasion, so they likely did not contribute substantially to the aerosol PM or settled dust present in climbing halls and no other RDCs were found in these items. Therefore, we concluded that another climbing related source must be the main contributor to the high concentrations of RDCs measured in settled dust and aerosol PM samples.

**Figure 4:**
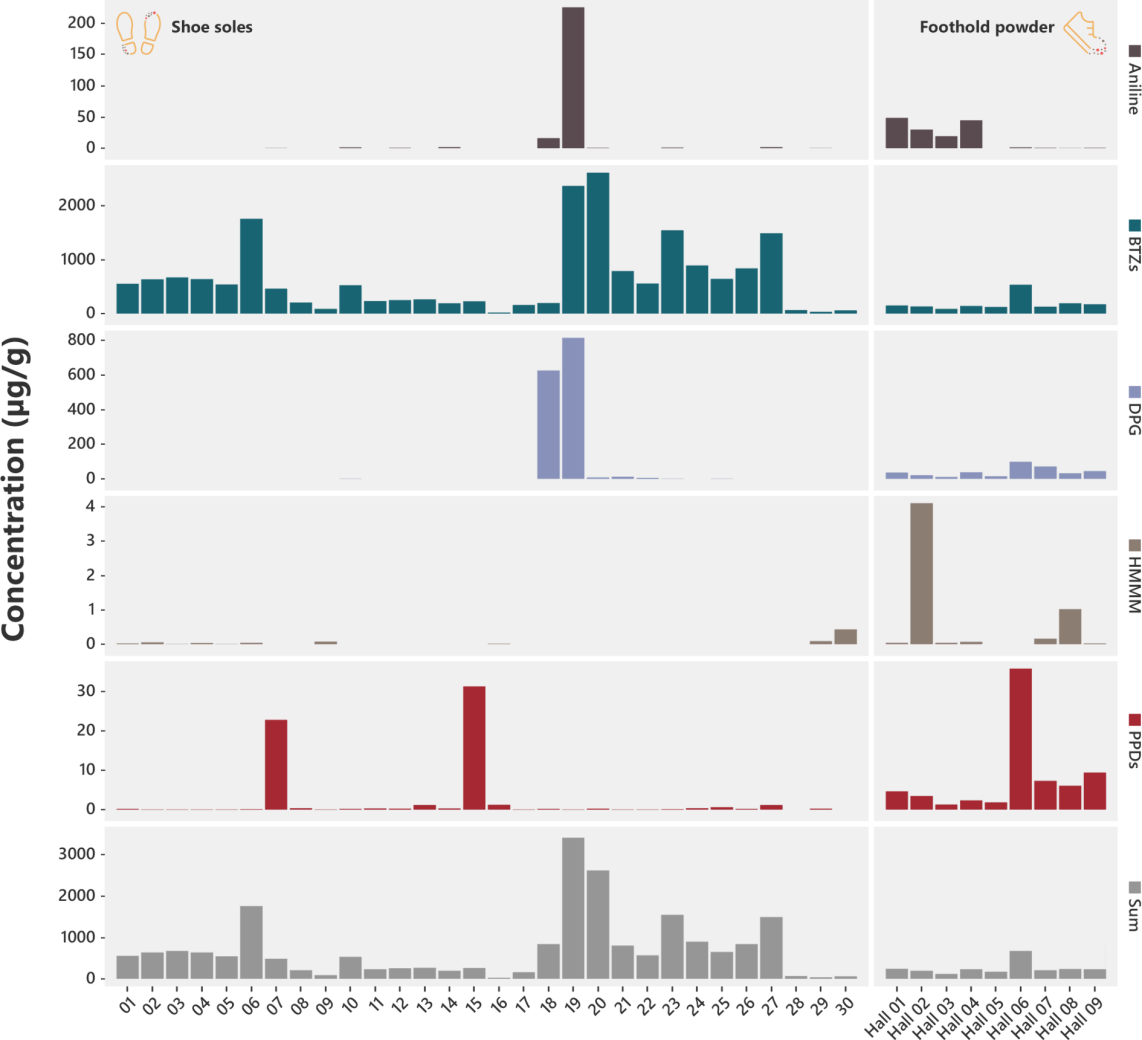
Rubber-derived compound concentrations in climbing shoes and foothold powder. RDC concentrations in 30 climbing shoe soles (left) and foothold powder from nine climbing halls (right). Concentrations of RDCs vary substantially among different shoe models. Foothold powder samples are representative of the variety of different shoe models.

All 15 RDCs were found in at least one of the 30 shoe sole samples screened. Concentrations were highly variable between shoe models with cumulative RDC concentrations ranging from 25 to 3,405 μg/g (mean: 711 μg/g) (Figure 4, Table S4). 2SH-BTZ was the main constituent (mean: 538 μg/g) representing on average 67% of the total mass of RDCs detected (Figure S3). BTZ, 2OH-BTZ and 2-amino-BTZ were detected in lower concentrations (mean: 58, 53, and 3 μg/g, respectively). As in other rubber products, 2SH-BTZ is likely used as a vulcanization accelerator during the curing process, while other benzothiazoles are typically considered to be impurities or degradation products.^7,44^ Unlike benzothiazoles, DPG and aniline were not detected in every shoe sole sample, with concentrations ranging from < LOQ to 814 μg/g and < LOQ to 225 μg/g, respectively. DPG is another vulcanization accelerator and may be used together with or instead of 2SH-BTZ (shoe soles 18 19; Figure 4). *p*-Phenylenediamine compounds were detected in most shoe sole samples in variable concentrations, with 6PPD and IPPD as the compounds with the highest concentrations (mean: 1303 ng/g and 661 ng/g, respectively). Of the numerous PPDs available, 6PPD and IPPD are the most commonly used rubber antiozonants.^45^ CPPD and DPPD were only detected sporadically and at trace levels (Table S4). The respective quinone transformation products, 6PPDq and IPPDq, were consistently detected (mean: 23 ng/g and 15 ng/g, respectively) and as expected, their concentration in the samples were correlated to the concentration of the parent compounds. CPPDq and DPPDq were also occasionally detected along with their parent compounds at very low concentrations (Table S4). Overall, RDC concentrations in shoe sole samples were highly variable and likely due to different compounding strategies used by manufacturers as well as the target product characteristics (i.e., stiffness, durability, performance, adhesiveness). RDC concentrations in shoe sole samples were generally lower (DPG and PPDs) or similar (benzothiazoles) to those in tire tread^7,44^ but higher than in other elastomeric consumer products.^7^

**Figure 5:**
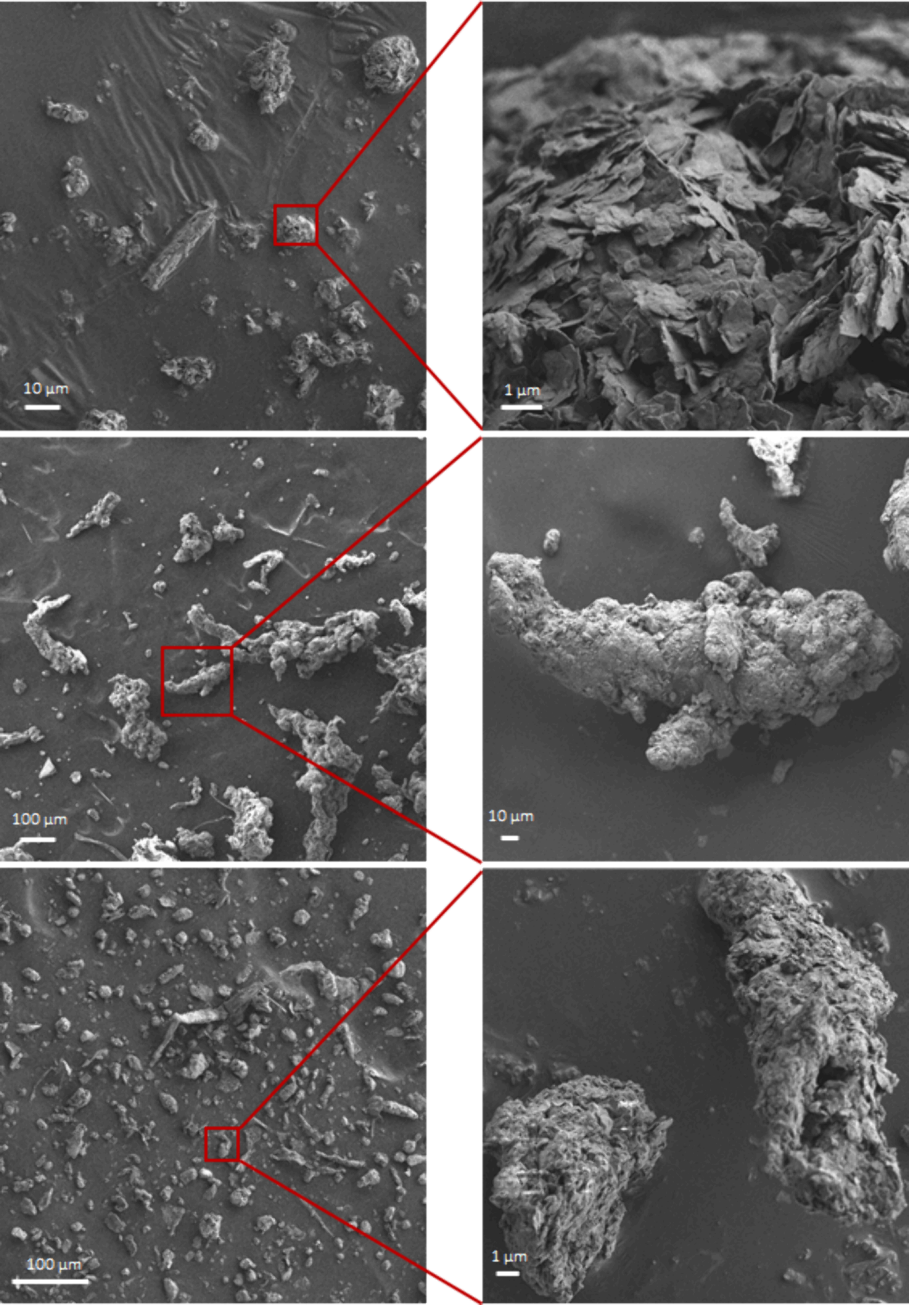
Scanning electron microscopy images of rubber particles in settled dust. Representative scanning electron microscopy images of (A) chalk (magnesium carbonate powder), (B) a foothold powder sample, and (C) a settled dust sample collected in climbing halls. Rubber particles resulting from the abrasion of shoe soles are visible in the foothold powder and were tentatively identified in the settled dust samples (B and C). Rubber particles can be distinguished from chalk particles (A) due to their elongated shape and surface physical characteristics with a smooth carbon-based surface compared to chalk. The elemental composition of a rubber particle was determined with energy dispersive X-ray and is shown in Figure S7. Surface roughness appears to increase between recently generated rubber particles identified in the foothold powder samples (B) and particles found in settled dust (C).

Based on visual inspection and SEM imaging, foothold powder collected from the top of several climbing footholds was comprised primarily of abraded climbing shoe soles, in contrast to settled dust samples, which was more heterogeneous in composition (Figures 1 and 5). Variability in RDC concentrations between triplicate foothold powder samples collected in one climbing hall was generally lower than for settled dust samples (Table S4), due to its homogeneity. However, some variability was observed, most likely due to dilution of the foothold powder samples with chalk or other dust. As these foothold powder (FP) samples were collected in public climbing halls, where visitors wear a variety of different climbing shoe models, these samples were highly representative of the variability found among individual shoe sole samples, both in terms of RDC concentrations and profile (Figures 4, S2). The abrasion of shoe soles generates fine, elongated rubber particles. These particles have the potential to become airborne over time, since it is common practice for climbers to brush particles off holds. SEM images of foothold powder confirmed that while some rubber particles are quite large (>100 μm) and unlikely to remain airborne, others fall within the <10 μm size range (Figure 5) and could remain airborne long enough to be inhaled. A detailed assessment of the particle size distribution of foothold powder was outside of the scope of this study. It has been previously shown that abrasion of particles and fibers containing additives drives the chemical composition of indoor dust,^46^ and it is highly likely that the fine rubber particles emitted via abrasion were captured by our air sampler. SEM imaging confirmed that elongated particles, visually very similar to those in foothold powder, were present in settled dust samples, including in the respirable size fraction (Figure 5). Based on the measurement of RDCs in shoe sole and foothold powder samples, images of elongated rubber particles in settled dust, and the lack of any alternative sources of RDCs in climbing halls, climbing shoes are most likely responsible for the elevated RDC concentrations in climbing hall air and settled dust.

### Transformations in Indoor Air Implicate Climbing Shoes as the Source of Rubber-Derived Compounds

All settled dust and aerosol PM (combined LRT and URT fractions) samples had RDC profiles which differed distinctly from shoe sole and foothold powder samples. Some target compounds were notably absent, or very reduced in the chemical profiles of aerosol PM and settled dust, due to their lack of detections in these samples (Figure 3, Table S4). For example, the mean fraction of 2SH-BTZ dropped from 48.0% in the foothold powder samples to 1.2% in the settled dust and aerosol PM samples. At the same time, the fractions of BTZ and 2OH-BTZ increased from 13.9% to 26.3% and 9.6% to 30.6%, respectively. Similarly, the fraction of 6PPD dropped from 1.6% in the foothold powder samples to 1.1% in the settled dust and aerosol PM samples, while the fraction of 6PPDq increased from 0.1% in the foothold powder samples to 4.7% in the settled dust and aerosol PM samples. The fraction of IPPD dropped from 0.8% to 0.4%, while IPPDq increased from 0.02% to 0.03% (Figure 3, S3).

All observed shifts (2SH-BTZ → 2OH-BTZ + BTZ; 6PPD → 6PPDq; IPPD → IPPDq) are likely the result of transformation reactions on particle surfaces. The rubber particles collected in aerosol PM samples must by virtue of the collection technique, exhibit small aerodynamic diameters with a correspondingly larger surface area. Rubber particles present in the collected settled dust samples were also enriched in smaller sized particulates after aerial transport (Figure 5). Such small particles have a high specific surface area, which allows for rapid reactions with reactive species in the surrounding gas phase. Ozone, as well as secondary species, such as the hydroxyl radical and NO_X_ drive chemical reactions on particle surfaces in indoor air.^47,48^ In contrast, the foothold powder samples exhibited a lower specific surface area because of the larger particle sizes (Figure 5) and, due to their continuous removal during climbing and brushing activity, as well as regular replacement of the climbing holds, they were too pristine to have undergone extensive transformation reactions prior to collection.

The chemical transformations observed here have been previously reported.^16,17,49,50^ To confirm that these transformations can also occur in rubber particles from climbing shoes, fast-aging experiments were conducted on foothold powder samples, using an ozone exposure chamber.^15^ After 4 h of exposure to a high ozone concentration (1 g/m^3^), the chemical profile of the foothold powder samples shifted substantially and corresponded to the chemical profile of the aerosol PM and settled dust samples (Figures S5, S6, Table S5). It is important to note that the ozone concentrations used in these experiments are significantly higher than what would typically be found in indoor climbing halls. Therefore, these experiments do not conclusively demonstrate that the observed transformations result from reactions with ozone, but they confirm that the RDCs in settled dust and aerosol PM samples are transformation products of those present in foothold powder. Given enough time, it is possible that the same transformations would occur in foothold powder at ambient ozone concentrations. However, it is unlikely that foothold powder remains on climbing holds long enough for this to happen.

Transformation of 2SH-BTZ is well studied in the aquatic environment,^51^ where 2OH-BTZ and BTZ are frequently reported as transformation products, including when transformation is induced by ozone.^49,50^ In our ozonation experiments, the mean concentration of 2SH-BTZ decreased from 78 μg/g to 23 μg/g (*p* < 0.05), while the mean concentrations of 2OH-BTZ and BTZ increased from 15 μg/g to 17 μg/g (*p* > 0.05), and 13 μg/g to 39 μg/g (*p* < 0.05) respectively. Likewise, it is well documented that PPDs react with ozone to form PPD-quinones,^16,17,52^ and the 6PPDq/6PPD ratio in crumb rubber has been related to the extent of environmental weathering.^7^ In our ozonation experiments, the mean 6PPDq/6PPD ratio increased from 0.04 to 0.12, and the mean IPPDq/IPPD ratio from 0.02 to 0.04. These changes were driven by decreasing 6PPD and IPPD concentrations, although the decreases were not statistically significant, while 6PPDq and IPPDq showed no significant changes in concentration. This likely reflects a balance between continuous formation and further transformation of 6PPDq and IPPDq, since it is known that 6PPDq is not a stable end product of 6PPD ozonation, but undergoes further transformations.^53^ The drops in 6PPD and IPPD concentrations likely indicate formation of many transformation products other than 6PPDq and IPPDq.^16,17,53^ Our experiments in conjunction with the body of literature reporting these ozone-induced transformations indicate that the observed shift of RDC profile in our samples results from atmospheric transformations of the RDCs after generation of the rubber particles on the climbing holds. This explanation is further supported by the fact that we did not find any significant alternative sources of the RDCs to aerosol PM or settled dust in climbing halls.

### Implications

In indoor climbing halls, concentrations of several RDCs substantially exceed previously reported values. Total daily intake of RDCs for individuals visiting or working in these facilities exceeds exposure via all other known routes. Indoor climbing is increasingly popular, with 6.36 million people participating in the sport in the US in 2023 alone,^54^ and climbing facilities worldwide likely employing many thousands of individuals who may be exposed to high RDC concentrations in the workplace. The occupational exposure of employees in the climbing shoe manufacturing sector also warrants further research.

The majority of the URT fraction of the collected aerosol PM would typically deposit in the nose and upper airways, and is subsequently swallowed.^55^ Aerosol PM from the LRT fraction has a higher probability of deposition within deeper regions of the lung.^56^ Thus, exposure to RDCs in climbing halls will be via both the gastrointestinal and respiratory systems. This study focused on the sampling of particle-associated RDCs and used an appropriate sampling device and analysis workflow. Due to the physicochemical characteristics of some RDCs (relatively high volatility and low octanol/air partition coefficient, Table S6), it is possible that some compounds would diffuse from the rubber particles and also be present in the gaseous phase in the air of climbing halls. In outdoor air, RDC partitioning was such that BTZ and 2OH-BTZ were mainly found in the gas phase (70 and 95%, respectively) but DPG, HMMM and all PPDs/PPDqs were mainly found in the particulate phase (>75%).^57^ If this partitioning also holds true in indoor environments, the overall (gas + particles) chemical burden in climbing halls air would substantially increase for BTZ and 2OH-BTZ and the human exposure to these RDCs would be higher than estimated here. It is well-known that indoor and outdoor partitioning differ, as the presence of many adsorptive materials such as mats, plastic holds, or indoor dust can enhance adsorption. In fact, surface area to volume ratios are typically 3 orders of magnitude higher in indoor than outdoor environments, which shifts partitioning to the particulate or surface deposition phase,^47^ resulting in lower concentrations of gaseous RDCs. Additionally, higher concentrations of organic compounds in indoor air compared to outdoor air also shift partitioning and enhance the probability of reactions with gaseous species.^47^ The behavior of RDCs and their fate in indoor air requires further investigations.

It is essential to investigate the leaching kinetics of RDCs from aerosol PM, ideally in epithelial lung fluid- and gastrointestinal fluid-mimetics. Further, the bioavailability of RDCs via different exposure routes, and their toxicity should be investigated in greater depth. Aerosol PM-bound PPDs and PPDqs may contribute to the oxidative potential of PM.^58^ Oxidative potential of aerosol PM induces oxidative stress and inflammation in the respiratory and cardiovascular systems.^59^ Indeed, organic tire extracts and tire wear particles have been shown to induce DNA damage, inflammation, and cell death in human lung cells.^8−10^

This study addressed only concentrations of particulate-associated RDCs, and did not account for gas phase concentrations. Sampling, particularly of airborne particulate matter, was limited to a few climbing halls in central Europe, and future studies should investigate whether RDC concentrations vary between climbing halls in different countries. While our study provided compelling evidence that atmospheric transformations shift rubber-derived compound profile in airborne particulate matter in climbing halls, the ozone concentrations used in our ozonation experiments were higher than those typically encountered in real-world conditions. It remains unclear whether ambient ozone and other reactive gas species concentrations could induce the observed transformations.

Future research and regulatory efforts aimed at identifying alternatives for toxic RDCs, such as 6PPD, must not overlook consumer products, such as climbing shoes, which contain a high additive content, and dominate the human exposure for a subset of the population. A recent study found that while RDC concentrations in a variety of rubber based consumer products are generally low, climbing shoes contain significantly higher levels.^7^ This contrast underscores that although rubber is widely used, only highly engineered consumer products, such as climbing shoes and tires, contain a high additive content. The observed variability in RDC concentrations in air across the five halls suggests that factors such as hall size, check-ins per hour, and ventilation may directly influence indoor air quality. Studies assessing the relationship between variables such as ventilation rate and RDC levels would help prioritize interventions to reduce RDC levels in climbing halls.

## Supporting Information Available

Supporting data (bouldering hall descriptors, extraction recovery, parameters used to calculate EDI_inh/ing_, ozonation experimental results, and physicochemical characteristics of RDCs), raw data used to generate all visuals (measured concentrations of all RDCs in all samples), supporting method descriptions and discussions (aerosol particulate matter sampling details, accelerated solvent extraction and UPLC-MS/MS method details, chemicals used, and blank and reference sample details), and supporting figures (literature comparison of background compounds, RDC profile in shoes and foothold powder, RDC concentration shifts, ozonation experiment results, and compound profile shifts) (PDF)
Additional data used to generate literature comparison figures (XLSX)
