# Supplementary material for: The Invisible Footprint of Climbing Shoes: High Exposure to Rubber Additives in Indoor Facilities
Source: ACS EST Air. 2025 Apr 24;2(5):930–42. doi: 10.1021/acsestair.5c00017 (PMC12070412; doi:10.1021/acsestair.5c00017)
Supplement: Supplementary file 1 — ea5c00017_si_001.pdf [file ea5c00017_si_001.pdf]

Supporting Information

The invisible footprint of climbing shoes: high exposure to rubber additives in indoor facilities

Anya Sherman[a,b,f] †, Thibault Masset[c]†, Lukas Wimmer[d,e], Leah K. Maruschka[a], Lea Ann Dailey[d], Thorsten Hüffer[a,f], Florian Breider[c], Thilo Hofmann[a,f*]

† These authors contributed equally.

a) University of Vienna, Centre for Microbiology and Environmental Systems Science, Environmental Geosciences EDGE, 1090 Vienna, Austria.

b) University of Vienna, Doctoral School in Microbiology and Environmental Science, 1090 Vienna, Austria.

c) EPFL – Ecole Polytechnique Fédérale de Lausanne, Central Environmental Laboratory, Institute of Environmental Engineering, ENAC, station 2, CH-1015 Lausanne, Switzerland

d) University of Vienna, Department of Pharmaceutical Sciences, 1090 Vienna, Austria.

e) University of Vienna, Doctoral School of Pharmaceutical, Nutritional and Sport Sciences, 1090 Vienna, Austria.

f) University of Vienna, Research Platform Plastics in the Environment and Society (PLENTY), 1090 Vienna, Austria.

*thilo.hofmann@univie.ac.at

Other Supplementary Materials for this manuscript include the following:

Source Data for Figures 1 and S1

Contents

Table S1: Bouldering Hall information 3

Supplementary Text S1: Aerosol particulate matter sampling details 4

Supplementary Text S2: Accelerated solvent extraction method details 6

Supplementary Text S3: Chemicals used 7

Supplementary Text S4: UPLC-MS/MS method details 8

Supplementary Text S5: Blank and reference samples 11

Table S2: Extraction Recovery 14

Table S3: Parameters used in equation (2) for calculation of $EDI_{inh/ing}$ 15

Table S4: Concentration of all rubber derived chemicals in all samples from every hall (n=9) and in shoe samples (n=30) 16

Figure S1: Literature comparison of RDCs in aerosol particulate matter and settled dust samples, which based on our data, do not necessarily arise uniquely from climbing activity. 22

Figure S2: RDC profile in all sample replicates from each hall 23

Figure S3: Rubber-derived compound profile in shoe soles and foothold powder 28

Figure S4: Rubber-derived compound concentration shifts. 29

Table S5: Ozonation Experimental Results 31

Figure S5: Ozonation experiment results 31

Figure S6: Rubber derived compound concentration shifts ozonation experiments 32

Figure S7: EDX imaging of a foothold powder particle. 34

Table S6: Physico-chemical characteristics of Rubber derived chemicals 36

Table S1: Bouldering Hall information

	Check-ins per hour	Ground area (m^2)	Hall age (years)	Climbing wall area (m^2)	Ventilation	Location
Hall 01	50.1	1200	7	N.A	Yes	Austria
Hall 02	37.7	Not available	19	1100	No	Austria
Hall 03	69.1	1100	4	1300	Yes	Austria
Hall 04	36.0	1100	7	1100	Yes	Austria
Hall 05	16.2	1300	3	1400	Yes	Austria
Hall 06	N.A	1800	N.A	N.A	Yes	France
Hall 07	N.A	2200	2	1500	No	Switzerland
Hall 08	N.A	N.A	N.A	1500	Yes	Switzerland
Hall 09	N.A	N.A	N.A	1000	Yes	Spain

Note: Not all data were available for all halls.

Supplementary Text S1: Aerosol particulate matter sampling details

Aerosol particulate matter (PM) is a complex mixture of airborne particles. Particles are classified by their aerodynamic diameter (AD), which takes the size, shape and density of the particles into account and therefore describes possible deposition patterns of airborne particles in different regions of the lung[1,2]. WHO differentiates between inhalable particles (AD < 100 µm) – which usually deposit in the nasopharyngeal region and are subsequently swallowed[3,4] – and respirable particles (AD < 10 µm), where the probability of particle deposition in the peripheral lung is dependent on AD[5,6]. WHO guidelines focus on $PM_{2.5}$ and PM_{10} which are defined as particles with an AD below 2.5 µm and 10 µm respectively[1]. They are of special interest because associations between short- and long-term exposure and adverse health effects have been shown[7–9].

First described by Hallworth & Westmoreland[10], the glass liquid impinger geometry and constant airflow of 60 ± 5 L/min, within the range of human respiration rates, leads to the separation of particles by their AD. Particles above 6.4 µm AD (upper respiratory tract - URT fraction) will impact at the mouth and throat piece or upper chamber, whereas particles below that size (lower respiratory tract - LRT fraction) will deposit in the lower chamber.

By introducing ceramic beads into the collection liquid of the lower chamber (granular fluid bed filtration), the entrapment efficiency of ultrafine particles increases[11]. During the current study, the liquid level was replenished regularly as water evaporation during the collection time has been shown to result in a poorer entrapment efficiency[12].

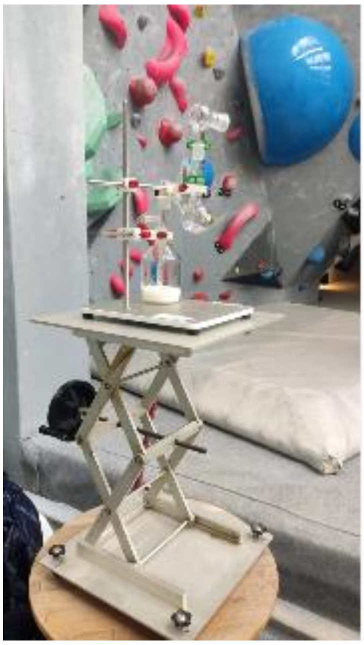

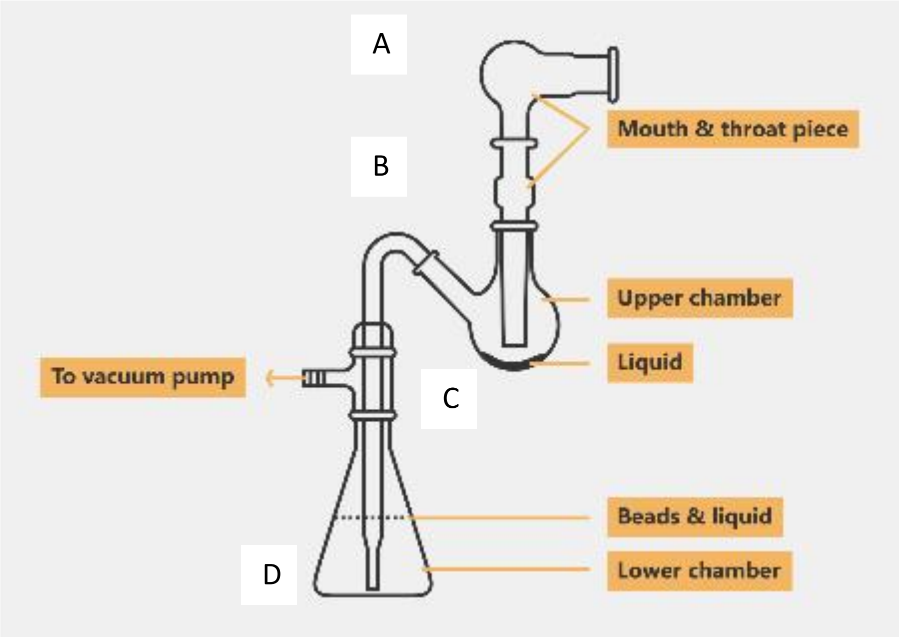

Compartment A & B: Mouth and throat piece (URT fraction part I)
Compartment C: Upper chamber (URT fraction part II)
Compartment D: Lower chamber (LRT fraction)

Vial preparation

Amber sample vials (250 mL) and liquid containers (Duran bottles, 250 mL and 500 mL) were muffled at 550°C for 5 h after removing all plastic parts. Vial lids were cleaned by rinsing three times with ethanol before closure and liquid containers were covered with tinfoil. For every day of sampling, a fresh bottle of MilliQ-water and ethanol (96% v/v, filtered 0.22 µm) was prepared to avoid contamination by ambient particles.

Sampling procedure

A glass liquid impinger was used as described in the European Pharmacopoeia (Ph. Eur.). Before the device was assembled, 7 mL of MilliQ-water was introduced into the upper chamber and 475 g (=130 mL) of ceramic beads (9730, ZY-P, 2.6-3.3 mm, SiLibeads, Sigmund Lindner GmbH, Warmensteinach, Germany) and 25 mL of MilliQ-water were introduced into the lower chamber (LRT fraction).

The device was elevated to place the mouthpiece at 142 cm above ground level and it was aligned to face the climbing wall at approximately 3m distance. Subsequently, the vacuum pump (LCP6, Copley Scientific Ltd., Nottingham, UK) was activated and the airflow was set to 60 ± 2 L/min, under constant monitoring using a flowmeter (5300-1, TSI Inc). During the sampling period of 3 h, the temperature, humidity and liquid levels of the upper and lower chambers were checked periodically (every 15 min) and liquids were replenished as needed. Collected material from multiple consecutive sampling periods at a single hall were combined during the washing procedure described below to be able to determine analytes of interest above the detection limit of the analytical method. In total, APM from 54 m^3 air was collected in each climbing hall (5 days × 3 h × 60 min × 60 L/min).

After switching off the pump, every part of the device was rinsed three times with 6 mL MilliQ-water and collected. The beads were rinsed with the wash fluid of the lower chamber, collected and subsequently washed three times more with 6 mL MilliQ-water. This procedure was repeated with ethanol. All rinse fluids were collected, whereby discrete vials for MilliQ water and ethanol were used, and stored at -20°C.

Based on the impinger geometry and air flow rate used (60 L/min), the collected aerosols could be separated into two fractions: 1) a fraction with predominantly extra-thoracic and tracheobronchial deposition based on the aerodynamic cut off > 6.4 µm (referred to as upper respiratory tract fraction; URT) and 2) a fraction with predominantly bronchial and alveolar deposition (aerodynamic cut off < 6.4 µm; referred to as lower respiratory tract fraction; LRT). The samples of the mouth and throat piece and the upper chamber were combined and represent the URT fraction of aerosol particulate matter; whereas the fluids from the lower chamber were collected in a separate vial to represent the LRT aerosol particulate matter fraction. Liquid was removed from the vials containing ethanol samples by rotary evaporation, whereas water was removed from the corresponding sample vials by lyophilization. The contents from the two dried vials corresponding to the upper chamber (i.e. water and ethanol wash) were collected in solvent and combined before extraction to obtain single values for the URT particulate matter concentration. Likewise, the contents from the two dried lower chamber vials (water and ethanol) were collected in solvent and combined before extraction to obtain single values for the LRT particulate matter concentrations (Tables 1-2, Table S2).

	**Sampling times**	**Average humidity**	**Average temperature**
**Hall 01**	17.5.2023 18:08-20:48	56%	19°C
	18.5.2023 17:23-20:26	65%	19°C
	19.5.2923 17:16-20:56	61%	18°C
	20.5.2023 17:25-20:29	62%	19°C
	21.5.2023 17:07-20:07	53%	21°C
**Hall 02**	23.04.2023 19:03-20:35	51%	21°C
	25.04.2023 16:48-21:05	52%	23°C
	26.04.2023 16:45-19:53	46%	22°C
	27.04.2023 17:03-20:59	43%	22°C
	28.04.2023 16:31-19:00	54%	20°C

**Hall 03**	15.04.2024 16:43-20:01	61%	22°C
	16.04.2024 16:55-21:06	58%	21°C
	17.04.2024 16:55-20:03	60%	22°C
	18.04.2024 17:21-20:29	54%	22°C
	19.04.2024 16:57-19:03	53%	22°C
**Hall 04**	22.04.2024 17:39-20:49	29%	19°C
	23.04.2024 17:16-20:29	37%	22°C
	24.04.2024 17:00-20:12	37%	22°C
	25.04.2024 17:01-20:15	34%	21°C
	26.04.2024 17:02-20:12	33%	22°C
**Hall 05**	29.04.2024 17:59-21:14	49%	21°C
	20.04.2024 16:53-19:59	50%	21°C
	01.05.2024 17:06-	54%	22°C
	02.05.2024 17:00-20:09	54%	22°C
	03.05.2024 17:13-19:17	55%	21°C

Cleaning

The glass liquid impinger was washed with MilliQ and ethanol after each day of sample collection and dried overnight before further use. Before sampling at the next location, the glass liquid impinger was cleaned thoroughly via sonication.

Supplementary Text S2: Accelerated solvent extraction method details

50 mg of all samples (shoe sole, foothold powder, settled dust and aerosol particulate matter samples) were transferred into an accelerated solvent extraction cell (ASE, Thermo-Fischer) filled with glass beads and internal standards were spiked. ASE extraction consisted of three static cycles of 5 minutes at 80°C using 100% acetonitrile as solvent. Extracts were concentrated under N_2 blowdown to approximately 0.5 mL, then reconstituted to 2 mL with acetonitrile, and filtered through 0.2 µm nylon filters before analysis.

Supplementary Text S3: Chemicals used

1. Vienna

Dichloromethane (99.9%) was purchased from Thermo Scientific. Acetonitrile (LCMS Grade) and ethanol (99.8%) were purchased from Sigma-Aldrich. Formic acid for HPLC was purchased from Merck.

IPPD, CPPD, DPPD, 6PPDq, IPPDq, DPPDq, DPPDq, and d5-6PPDq were from HPC Standards®. DPG, benzothiazole, aniline, 2-mercaptobenzothiazole, 2-hydroxybenzothiazole, 2-aminobenzothiazole, and 6PPD were purchased from Sigma-Aldrich. HMMM was purchased from TCI Europe. D4-Benzothiazole was purchased from LGC Standards.

2.Lausanne

Acetonitrile was purchased from Biosolve® and dichloromethane were from Carlo Erba®. All solvents were UPLC grades.

Standards of aniline, Aniline d5, benzothiazole, Benzothiazole-d4, 2-hydroxybenzothiazole, 2-mercaptobenzothiazole were from Sigma-aldrich®. 1,3-diphenylguanidine and 1,3 diphenylurea-d10 were from Chemie Brunschwig AG®. Hexa(methoxymethyl)melamine, 6PPD, IPPD, CPPD, DPPD, 6PPDq, IPPDq, CPPDq and DPPDq were from HPC Standards®. Standards quality was always >95%

Supplementary Text S4: UPLC-MS/MS method details

All samples were measured with ultra-high pressure liquid chromatography coupled with triple quadrupole mass spectrometry, in either Vienna Austria or Lausanne Switzerland. Below are the method details as used in each laboratory.

1.1 Vienna

All samples measured in Vienna were analyzed by ultra-performance liquid chromatography - triple quadrupole mass spectrometry (Agilent 1290 Infinity II - Agilent 6470) using a C18 column (Acquity HSS T3, 1.8 µm, Waters) in the positive ionization mode (capillary voltage: 2500 V) via multiple reaction monitoring (MRM). The LC flow rate was 0.6 mL/min at a column temperature of 40 °C and the injection volume was 5 µL. The mobile phase consisted of ultrapure water (Phase A) and acetonitrile (Phase B), both containing 0.1% formic acid. The eluent gradient was held at 95% Phase A for one minute, then set that the contribution of Phase A decreased from 95 to 5% over 9 min, was held at 5% for 2 min and increased again to 95% over the last 1 min. Column was then re-equilibrated at 95% Phase A for one minute. Electrospray ionization (ESI) was achieved at gas temp 240ºC, gas flow 5 L min^{-1}. The nebulizer was set to 30 psi; sheath gas to 250ºC, 11 L min^{-1}. Quantification was achieved by external calibration standards prepared in acetonitrile (0.01 to 500 µg L^{-1}). Transitions from precursor ions to product ions are specified in the following table. At least 2 product ions were monitored per compound, quantifier ions are indicated in bold.

Compound name	ISTD	Precursor ion m/z	Product ion m/z	Fragmentor voltage (V)	Collision energy (V)	Cell accelerator voltage (V)	RT (min)
**2-aminobenzothiazole**	**d4-BTZ**	**151**	**109**	**150**	**30**	**5**	**5.4**
2-aminobenzothiazole		151	65	150	38	5	5.4
**2-hydroxybenzothiazole**	**d4-BTZ**	**152**	**124**	**140**	**22**	**4**	**3.6**
2-hydroxybenzothiazole		152	92	140	26	4	3.6
**2-mercaptobenzothiazole**	**d4-BTZ**	**168**	**135**	**135**	**28**	**5**	**3.1**
2-mercaptobenzothiazole		168	124	135	24	5	3.1
2-mercaptobenzothiazole		168	109	135	28	5	3.1
**6PPD**	**d5-6PPD-q**	**269**	**184**	**150**	**45**	**5**	**3.8**
6PPD		269	107	150	45	5	3.8
6PPD		269	93	150	45	5	3.8
6PPD-quinone		299	241	150	53	5	5.8
6PPD-quinone		299	215	150	30	5	5.8
**6PPD-quinone**	**d5-6PPD-q**	**299**	**187**	**150**	**30**	**5**	**5.8**
**Aniline**	**d4-BTZ**	**94**	**77**	**100**	**22**	**4**	**2.6**
Aniline		94	51	100	23	4	2.6
Aniline		94	50	100	41	4	2.6
**Benzothiazole**	**d4-BTZ**	**136**	**109**	**150**	**31**	**5**	**2.8**
Benzothiazole		136	77	150	27	5	2.8
Benzothiazole		136	65	150	38	5	2.8
Benzothiazole-d4		140	113	150	31	5	4.5
Benzothiazole-d4		140	81	150	19	5	4.5
**Benzothiazole-d4**		**140**	**69**	**150**	**36**	**5**	**4.5**
**CPPD**	**d5-6PPD-q**	**267.2**	**184**	**100**	**30**	**5**	**3.8**
CPPD		267.2	107	100	50	5	3.8
CPPD		267.2	93.1	100	46	5	3.8

CPPD-quinone		297.1	215	130	18	5	6
CPPD-quinone		297.1	187	130	34	5	6
**CPPD-quinone**	**d5-6PPD-q**	**297.1**	**55.2**	**130**	**50**	**5**	**6**
**d5-6PPD-quinone**		**304.2**	**246.1**	**110**	**36**	**4**	**6.5**
d5-6PPD-quinone		304.2	220.1	110	36	5	6.5
d5-6PPD-quinone		304.2	192.1	110	36	5	6.5
DPG		212	195	150	20	5	1.8
**DPG**	**d5-6PPD-q**	**212**	**119**	**150**	**20**	**5**	**1.8**
DPG		212	94	150	20	5	1.8
DPPD		260.1	183	130	38	5	6.4
**DPPD**	**d5-6PPD-q**	**260.1**	**167**	**130**	**46**	**5**	**6.4**
DPPD		260.1	156	130	46	5	6.4
**DPPD-quinone**	**d5-6PPD-q**	**291.1**	**263**	**150**	**22**	**5**	**5.5**
DPPD-quinone		291.1	235.2	150	34	5	5.5
DPPD-quinone		291.1	144	150	38	5	5.5
HMMM		391	283	150	15	5	3.5
HMMM		391	253	150	23	5	3.5
HMMM		391	207	150	19	5	3.5
**HMMM**	**d5-6PPD-q**	**391**	**177**	**150**	**35**	**5**	**3.5**
**IPPD**	**d5-6PPD-q**	**227.2**	**184**	**100**	**18**	**5**	**3**
IPPD		227.2	118	100	46	5	3
IPPD		227.2	107	100	46	5	3
IPPD-quinone		257.2	216	110	30	5	4.8
**IPPD-quinone**	**d5-6PPD-q**	**257.2**	**187**	**110**	**30**	**5**	**4.8**
IPPD-quinone		257.2	170	110	34	5	4.8

1.2 Lausanne

UPLC-MSMS analyses

UPLC-MSMS analyses were carried out with an ACQUITY UPLC system that was coupled to a Xevo TQ MS mass spectrometer equipped with an electrospray ionization source (ESI) (Waters®). Chromatographic separation was performed with an ACQUITY UPLC HSS T3 column (100 × 2.1 mm, 1.8 µm) at a flow rate of 0.40 mL min^{-1} and a column temperature of 30 °C. The mobile phase consisted of (A) 0.1 % formic acid in 95 % water and 5 % acetonitrile and (B) 0.1 % formic acid in 95 % acetonitrile and 5 % water. The elution gradient used is presented in table 1.

Step	Time (min)	Flow (mL/min)	%A	%B
1	0.00	0.40	95.0	5.0
2	0.50	0.40	95.0	5.0
3	5.00	0.40	5.0	95.0
4	9.00	0.40	5.0	95.0
5	10.00	0.40	95.0	5.0
6	12.00	0.40	95.0	5.0

Table 1: Elution gradient of the liquid chromatographic column with solvents A: 0.1 % formic acid in 95 % water and 5 % acetonitrile and B: 0.1 % formic acid in 95 % acetonitrile and 5 % water

The MS parameters were set as follows; electrospray in positive mode, source temperature: 150°C, desolvation temperature 600°C, cone gas flow (N_2): 50 L/h, desolvation gas flow: 1000 L/h, collision gas flow: 0.15 mL /min, ion spray voltage: 1500 V, cone voltage: 30 V.

The optimal instrumental parameters for each analyte were obtained by tuning using direct infusion. Quantitative analyses were performed in MS/MS mode. Identification of the analytes was based on comparison of two MRM transitions and retention times. The analyte specific parameters are presented in Table 2. Data was acquired and processed with MassLynx V4.2 (Waters®).

Compound	RT (min)	Parent ion (m/z)	Daughter ion (m/z)	Daughter Ion	Dwell time (s)	Cone voltage (V)	Collision voltage (V)
Aniline	0.97	94.10	76.96	Confirmation	0.078	32	16
			94.01	Quantifier			
Aniline d5	0.97	98.87	54.32	Confirmation	0.078	36	22
			82.00	Quantifier			
Diphenylguanidine	2.50	211.97	93.98	Confirmation	0.077	30	20
			119.02	Quantifier			
Benzothiazole	3.53	136.10	64.93	Confirmation	0.031	40	22
			108.90	Quantifier			
Benzothiazole d4	3.52	139.86	68.83	Confirmation	0.031	44	24
			112.31	Quantifier			
2-hydroxybenzothiazole	3.28	152.10	79.94	Confirmation	0.031	44	21
			123.96	Quantifier			
2-mercaptobenzothiazole	3.68	168.03	91.97	Confirmation	0.031	36	21
			135.08	Quantifier			
6PPD	3.71	269.05	106.99	Confirmation	0.050	24	42
			184.09	Quantifier			
Diphenylurea d10	4.16	222.95	81.17	Confirmation	0.050	28	28
			99.24	Quantifier			
6PPD Quinone	5.37	299.00	187.00	Confirmation	0.050	28	30
			241.00	Quantifier			
6PPD Quinone d5	5.35	304.01	192.04	Quantifier	0.050	28	28
HMMM	3.50	391.28	177.05	Confirmation	0.01	12	6
			359.22	Quantifier			
IPPD	3.01	227.16	107.04	Confirmation	0.05	25	38
			184.13	Quantifier			
IPPD-Q	4.33	257.14	187.12	Confirmation	0.04	28	23
			215.08	Quantifier			
CPPD	3.50	267.20	92.90	Confirmation	0.05	24	16
			184.80	Quantifier			
CPPD-Q	5.12	297.20	187.12	Confirmation	0.03	30	16
			215.08	Quantifier			
DPPD	5.28	260.14	167.16	Confirmation	0.03	38	30
			183.09	Quantifier			
DPPD-Q	4.80	291.14	143.90	Confirmation	0.03	32	20
			263.12	Quantifier			

Supplementary Text S5: Blank and reference samples

Collection blanks were collected before each composite sampling event. The glass liquid impinger was assembled in the climbing hall (without air flow), then immediately rinsed with exactly the same protocol as the samples. Storage blanks were also prepared in the climbing hall by filling amber glass storage vials with MilliQ-water or ethanol, and then opening and closing the vials five times, to simulate the collection of samples. Collection and storage blanks were stored and extracted in the same manner as aerosol particulate matter samples. Laboratory blanks were prepared in the same manner as samples, beginning with accelerated solvent extraction. The total mass of RDCs measured in collection blanks (representing contamination accumulated during sample collection, storage, and laboratory processing) was subtracted from the mass of RDCs measured in corresponding aerosol particulate matter samples.

In the following tables, the terms URT and LRT fractions are used to denote samples where aerosols were actively collected from the air sampling devices, while the terms upper and lower chambers are used when blank measurements were collected from the device without active air sampling. Total particle mass in collection and storage blanks was determined gravimetrically. In the blanks taken for the upper and lower chambers of the air sampling device in each climbing hall, particle masses ranging from –3.9 to 0.3 mg were detected, reflecting the error of the analytical balance.

Total particles detected:

Blank	Particle Mass (mg)
Storage blank	-3.9
Collection blank – Hall 01 upper chamber	-2.4
Collection blank – Hall 01 lower chamber	-2.9
Collection blank – Hall 02 upper chamber.	0.3
Collection blank – Hall 02 lower chamber	-1.7
Collection blank – Hall 03 upper chamber	1.9
Collection blank – Hall 03 lower chamber	0.0
Collection blank – Hall 04 upper chamber	2.8
Collection blank – Hall 04 lower chamber	1.8
Collection blank – Hall 05 upper chamber	0.5
Collection blank – Hall 05 lower chamber	7.2

Name	LOQ	Laboratory blanks				Storage blank	Collection blanks			
						Hall 1	Upper chamber hall 1	Upper chamber hall 2	Lower chamber hall 1	Lower chamber hall 2
**Aniline**	50	<LOQ	<LOQ	<LOQ	<LOQ	<LOQ	<LOQ	<LOQ	<LOQ	<LOQ
**DPG**	0.73	<LOQ	<LOQ	<LOQ	<LOQ	1.3	<LOQ	1.0	0.88	2.81
**2OH-BTZ**	3.7	78.2	<LOQ	<LOQ	<LOQ	6.3	9.3	10.5	25.9	20.8
**IPPD**	0.55	<LOQ	<LOQ	<LOQ	<LOQ	0.7	0.55	0.81	0.81	0.63
**BTZ**	20	<LOQ	<LOQ	<LOQ	<LOQ	35	42.9	43.4	45.4	50.6
**2amino-BTZ**	150	<LOQ	<LOQ	<LOQ	<LOQ	<LOQ	<LOQ	<LOQ	<LOQ	<LOQ
**2SH-BTZ**	29.5	1621.6	<LOQ	<LOQ	<LOQ	<LOQ	32.4	30.2	<LOQ	<LOQ
**HMMM**	0.2	<LOQ	<LOQ	<LOQ	<LOQ	<LOQ	<LOQ	<LOQ	<LOQ	<LOQ
**CPPD**	0.2	<LOQ	<LOQ	<LOQ	<LOQ	<LOQ	<LOQ	<LOQ	<LOQ	<LOQ
**6PPD**	2.9	<LOQ	<LOQ	<LOQ	<LOQ	<LOQ	<LOQ	<LOQ	<LOQ	<LOQ
**IPPDq**	0.2	<LOQ	<LOQ	<LOQ	<LOQ	<LOQ	<LOQ	<LOQ	<LOQ	<LOQ
**DPPDq**	5.9	<LOQ	<LOQ	<LOQ	<LOQ	<LOQ	<LOQ	<LOQ	<LOQ	<LOQ
**6PPDq**	0.2	<LOQ	<LOQ	<LOQ	<LOQ	<LOQ	<LOQ	<LOQ	<LOQ	<LOQ
**CPPDq**	2	<LOQ	<LOQ	<LOQ	<LOQ	<LOQ	<LOQ	<LOQ	<LOQ	<LOQ
**DPPD**	5	<LOQ	<LOQ	<LOQ	<LOQ	<LOQ	<LOQ	<LOQ	<LOQ	<LOQ

Name	Collection blanks			
	Upper chamber hall 3	Lower chamber hall 3	Upper chamber hall 4	Lower chamber hall 4
**Aniline**	<LOQ	<LOQ	<LOQ	<LOQ
**DPG**	<LOQ	<LOQ	<LOQ	<LOQ
**2OH-BTZ**	<LOQ	16.3	32.5	<LOQ
**IPPD**	<LOQ	2.28	<LOQ	<LOQ
**BTZ**	<LOQ	<LOQ	<LOQ	<LOQ
**2amino-BTZ**	<LOQ	<LOQ	<LOQ	<LOQ
**2SH-BTZ**	<LOQ	<LOQ	<LOQ	<LOQ
**HMMM**	<LOQ	<LOQ	<LOQ	<LOQ
**CPPD**	<LOQ	<LOQ	<LOQ	<LOQ
**6PPD**	<LOQ	<LOQ	<LOQ	<LOQ
**IPPDq**	<LOQ	<LOQ	<LOQ	<LOQ
**DPPDq**	<LOQ	<LOQ	<LOQ	<LOQ
**6PPDq**	<LOQ	4.12	<LOQ	<LOQ
**CPPDq**	<LOQ	<LOQ	<LOQ	<LOQ
**DPPD**	<LOQ	<LOQ	<LOQ	<LOQ

Rubber-derived compounds in reference samples from an office and investigated alternative sources (ng).

Name	LOQ	Mat-fiber	Mat-smooth	Hold	Reference sample URT fraction	Reference sample LRT fraction
**Aniline**	50	<LOQ	<LOQ	<LOQ	<LOQ	<LOQ
**DPG**	0.73	<LOQ	<LOQ	<LOQ	<LOQ	<LOQ
**2OH-BTZ**	3.7	<LOQ	<LOQ	<LOQ	<LOQ	<LOQ
**IPPD**	0.55	<LOQ	4.75	3.05	14.2	32.2
**BTZ**	20	<LOQ	<LOQ	<LOQ	<LOQ	<LOQ
**2amino-BTZ**	150	<LOQ	<LOQ	<LOQ	<LOQ	<LOQ
**2SH-BTZ**	29.5	<LOQ	<LOQ	<LOQ	<LOQ	<LOQ
**HMMM**	0.2	<LOQ	<LOQ	<LOQ	<LOQ	<LOQ
**CPPD**	0.2	<LOQ	<LOQ	<LOQ	<LOQ	<LOQ
**6PPD**	2.9	<LOQ	<LOQ	<LOQ	<LOQ	5.2
**IPPDq**	0.2	<LOQ	0.33	<LOQ	<LOQ	<LOQ
**DPPDq**	5.9	<LOQ	<LOQ	<LOQ	<LOQ	<LOQ
**6PPDq**	0.2	<LOQ	<LOQ	<LOQ	<LOQ	<LOQ
**CPPDq**	2	<LOQ	<LOQ	<LOQ	<LOQ	<LOQ
**DPPD**	5	<LOQ	<LOQ	<LOQ	<LOQ	<LOQ

Table S2: Extraction Recovery

Recovery tests were performed by both research groups involved in the study (Lausanne and Vienna) to assess the inter-lab variability of the extraction method used (see Material and Methods section). Recovery tests were conducted by spiking target compounds into an empty (without sample) ASE cell and extracting and measuring as described in the Materials and Methods section. As additive-free sample matrices (shoe soles or dust) do not exist, it was not possible to conduct recovery tests with actual sample matrices. To account for this limitation, deuterated internal standards were spiked to the samples before extraction, which account for extraction efficiency and matrix effects. All concentrations are corrected according to deuterated internal standard recoveries. Absolute recovery is the measured concentration as a percentage of the spiked concentration. Relative recovery is the absolute recovery normalized by its respective internal standard. In Vienna, target compounds were spiked at 500 ng/mL (benzothiazoles) or 100 ng/mL (all other compounds), to mimic the concentrations observed in most samples.

**Compound**	**Extraction recovery (%) (mean ± sd)** Lausanne	**Extraction recovery (%) (mean ± sd)** Vienna	
	relative	absolute	relative
Aniline	81 ± 8	62 ± 29	66 ± 22
BTZ	75 ± 14	96 ± 3	98 ± 4
2amino-BTZ	91 ± 3	100 ± 4	98 ± 4
2OH-BTZ	96 ± 3	87 ± 4	89 ± 5
2SH-BTZ	42 ± 23	29 ± 11	32 ± 14
6-PPDq	90 ± 7	73 ± 24	97 ± 6
DPG	82 ± 15	65 ± 26	76 ± 20
6PPD	57 ± 44	73 ± 20	87 ± 10
HMMM	109 ± 10	107 ± 5	145 ± 34
CPPD	32 ± 27	82 ± 23	101 ± 7
CPPDq	92 ± 5	78 ± 16	109 ± 9
IPPD	33 ± 31	77 ± 19	90 ± 9
IPPDq	91 ± 4	84 ± 11	117 ± 18
DPPD	67 ± 23	102 ± 21	132 ± 7
DPPDq	86 ± 36	62 ± 27	74 ± 21

Table S3: Parameters used in equation (1) for calculation of $EDI_{inh/ing}$. IR values were determined for low intensity activities (employees) and moderate intensity (adult climbers) for individuals aging 21 – 31 yrs according to US EPA[13]. Calculations were performed for employees working 8h/day, 5days/week and for adult climbers visiting the facilities for 3h/day, 3 days/week. IR = Inhalation rate, ET = exposure time per day, EF = exposure frequency, BW = average body weight, Cf = conversion factor from year to day.

	Employees	Adult climbers
IR (m^3/day)	17.3	37.4
ET (h/day)	8	3
EF (day/years)	228	156
BW (kg)	63	63
Cf	365	365

Table S4: Concentration of all rubber derived chemicals in all samples from every hall (n=9) and in shoe samples (n=30)

Hall 01 (ng/g)	FP A	FP B	FP C	SD A	SD B	SD C	URT APM	LRT APM
**Aniline**	38700	61650	45670	531	307	311	1681	1020
**DPG**	36190	45410	27970	329	115	183	3117	790
**2OH-BTZ**	30370	29580	29360	479	1155	1199	1990	1515
**IPPD**	888	1556	417	6.59	<LOQ	14.3	69.0	26.1
**BTZ**	1963	21780	21030	<LOQ	<LOQ	925	3354	1927
**2amino-BTZ**	476	425	376	47.9	64.0	142	<LOQ	<LOQ
**2SH-BTZ**	79880	102100	142600	<LOQ	<LOQ	<LOQ	<LOQ	<LOQ
**HMMM**	30.9	51.3	46.9	<LOQ	52.7	96.7	56.0	31.9
**CPPD**	13.8	16.3	10.9	<LOQ	<LOQ	<LOQ	<LOQ	<LOQ
**6PPD**	4677	2978	2401	<LOQ	<LOQ	<LOQ	93.0	54.5
**IPPDq**	30.9	54.7	24.2	<LOQ	<LOQ	<LOQ	4.78	5.58
**DPPDq**	<LOQ	<LOQ	<LOQ	<LOQ	<LOQ	<LOQ	<LOQ	<LOQ
**6PPDq**	293	279	201	179	312	307	316	148
**CPPDq**	<LOQ	<LOQ	<LOQ	<LOQ	<LOQ	<LOQ	<LOQ	<LOQ
**DPPD**	<LOQ	<LOQ	<LOQ	<LOQ	<LOQ	<LOQ	<LOQ	<LOQ

Hall 02 (ng/g)	FP A	FP B	FP C	SD A	SD B	SD C	URT APM	LRT APM
**Aniline**	38360	18800	32650	884	<LOQ	<LOQ	2804	1459
**DPG**	33430	12470	21380	721	203	58.0	4539	2182
**2OH-BTZ**	25410	12980	25870	1086	1262	1063	4112	1419
**IPPD**	512	375	832	7.98	8.25	6.91	133	56.0
**BTZ**	18610	8674	17430	1190	757	<LOQ	13890	2746
**2amino-BTZ**	326	155	317	40.4	60.3	52.5	<LOQ	<LOQ
**2SH-BTZ**	95560	77900	116400	<LOQ	<LOQ	<LOQ	1409	<LOQ
**HMMM**	324	6642	5326	441	17000	15180	498	275
**CPPD**	7.19	16.8	16.3	<LOQ	<LOQ	<LOQ	<LOQ	<LOQ
**6PPD**	1493	1379	4849	30.5	<LOQ	<LOQ	305	116
**IPPDq**	35.4	17.2	56.6	<LOQ	<LOQ	<LOQ	42.5	21.2
**DPPDq**	<LOQ	<LOQ	<LOQ	<LOQ	<LOQ	<LOQ	<LOQ	<LOQ
**6PPDq**	224	115	326	267	206	190	714	408
**CPPDq**	<LOQ	<LOQ	<LOQ	<LOQ	<LOQ	<LOQ	<LOQ	<LOQ
**DPPD**	<LOQ	<LOQ	<LOQ	<LOQ	<LOQ	<LOQ	<LOQ	<LOQ

Hall 03 (ng/g)	FP A	FP B	FP C	SD A	SD B	SD C	URT APM	LRT APM
**Aniline**	17630	26450	14920	412	1424	1185	<LOQ	<LOQ

**DPG**	11980	12590	11750	297	1235	793	40.8	36.4
**2OH-BTZ**	11610	16000	14530	1019	1471	1443	1430	4075
**IPPD**	114	191	357	8.45	10.6	<LOQ	71.6	24.9
**BTZ**	7568	14060	10610	861	1477	2825	1326	1393
**2amino-BTZ**	224	212	249	47.7	62.2	70.2	<LOQ	<LOQ
**2SH-BTZ**	51910	72690	67060	<LOQ	<LOQ	<LOQ	581	509
**HMMM**	39.0	50.5	42.3	178	186	111	76.2	118
**CPPD**	<LOQ	5.94	10.0	<LOQ	<LOQ	<LOQ	<LOQ	<LOQ
**6PPD**	1069	612	1106	<LOQ	31.1	<LOQ	125	224
**IPPDq**	4.03	4.60	6.34	<LOQ	<LOQ	<LOQ	<LOQ	<LOQ
**DPPDq**	<LOQ	<LOQ	<LOQ	<LOQ	<LOQ	<LOQ	<LOQ	<LOQ
**6PPDq**	134	130	124	371	323	405	118	51.5
**CPPDq**	<LOQ	<LOQ	<LOQ	<LOQ	<LOQ	<LOQ	<LOQ	<LOQ
**DPPD**	<LOQ	<LOQ	<LOQ	<LOQ	<LOQ	<LOQ	<LOQ	<LOQ

**Hall 04 (ng/g)**	**FP A**	**FP B**	**FP C**	**SD A**	**SD B**	**SD C**	**URT APM**	**LRT APM**
**Aniline**	39640	61250	33220	1329	389	629	<LOQ	<LOQ
**DPG**	26270	61430	28350	855	262	362	<LOQ	5.98
**2OH-BTZ**	27270	24550	23970	1499	<LOQ	845	566	1207
**IPPD**	255	364	471	20.5	11.3	12.1	<LOQ	<LOQ
**BTZ**	20450	22840	18550	2497	788	1122	872	<LOQ
**2amino-BTZ**	290	311	354	73.3	44.6	48.6	<LOQ	<LOQ
**2SH-BTZ**	85890	98470	108400	<LOQ	<LOQ	<LOQ	<LOQ	<LOQ
**HMMM**	60.7	92.7	67.7	178	58.5	86.3	<LOQ	<LOQ
**CPPD**	7.75	12.0	7.60	<LOQ	<LOQ	<LOQ	<LOQ	<LOQ
**6PPD**	1550	1971	1600	48.6	38.7	43.3	67.9	<LOQ
**IPPDq**	23.6	26.1	21.0	8.17	<LOQ	<LOQ	<LOQ	<LOQ
**DPPDq**	<LOQ	<LOQ	<LOQ	46.8	<LOQ	<LOQ	<LOQ	<LOQ
**6PPDq**	233	241	163	356	219	576	60.0	20.7
**CPPDq**	<LOQ	<LOQ	<LOQ	<LOQ	<LOQ	<LOQ	<LOQ	<LOQ
**DPPD**	<LOQ	<LOQ	<LOQ	<LOQ	<LOQ	<LOQ	<LOQ	<LOQ

**Hall 05 (ng/g)**	**FP A**	**FP B**	**SD A**	**SD B**	**SD C**
**Aniline**	<LOQ	20910	<LOQ	526	<LOQ
**DPG**	15370	15990	137	431	168
**2OH-BTZ**	<LOQ	19890	812	857	642
**IPPD**	238	678	6.60	10.8	9.60
**BTZ**	<LOQ	16030	813	964	1136
**2amino-BTZ**	573	316	37.5	44.1	32.4
**2SH-BTZ**	128100	118900	<LOQ	<LOQ	<LOQ

**HMMM**	<LOQ	49.5	53.3	85.8	102
**CPPD**	<LOQ	14.0	<LOQ	<LOQ	<LOQ
**6PPD**	901	1430	<LOQ	<LOQ	<LOQ
**IPPDq**	<LOQ	11.2	<LOQ	<LOQ	<LOQ
**DPPDq**	<LOQ	<LOQ	<LOQ	<LOQ	<LOQ
**6PPDq**	308	88.6	280	323	298
**CPPDq**	<LOQ	<LOQ	<LOQ	<LOQ	<LOQ
**DPPD**	<LOQ	<LOQ	<LOQ	<LOQ	<LOQ

**Hall 06 (ng/g)**	**FP A**	**FP B**	**FP C**	**SD A**	**SD B**	**SD C**
**Aniline**	1237	1633	1906	237	659	493
**DPG**	118000	93820	87500	4463	4927	9652
**2OH-BTZ**	41090	33310	42970	8945	9502	6879
**IPPD**	22590	7439	8907	163	384	307
**BTZ**	73030	59430	66550	6398	34450	32390
**2amino-BTZ**	627	478	527	131	105	86.3
**2SH-BTZ**	553000	357000	383800	542	4369	2294
**HMMM**	<LOQ	<LOQ	<LOQ	36.9	57.9	33.9
**CPPD**	167	67.8	90.9	<LOQ	<LOQ	<LOQ
**6PPD**	33840	13640	19350	30.8	178	165
**IPPDq**	159	96.9	96.3	<LOQ	<LOQ	41.7
**DPPDq**	<LOQ	<LOQ	<LOQ	<LOQ	<LOQ	<LOQ
**6PPDq**	299	185	324	23.7	91.5	73.5
**CPPDq**	<LOQ	<LOQ	<LOQ	<LOQ	<LOQ	<LOQ
**DPPD**	27.2	12.0	15.8	0.40	<LOQ	0.80

**Hall 07 (ng/g)**	**FP A**	**FP B**	**FP C**	**SD A**	**SD B**	**SD C**
**Aniline**	744	861	740	232	797	262
**DPG**	69410	82330	65950	4053	2223	3743
**2OH-BTZ**	14400	15030	14080	1394	4021	1340
**IPPD**	1429	1660	3604	20.8	<LOQ	18.1
**BTZ**	40940	36820	35140	<LOQ	3243	3206
**2amino-BTZ**	457	715	429	73.0	171	40.0
**2SH-BTZ**	74020	112000	43480	<LOQ	<LOQ	<LOQ
**HMMM**	92.4	231	160	207	2224	333
**CPPD**	11.6	13.4	10.2	2.10	7.60	<LOQ
**6PPD**	3712	3302	7355	273	33.3	62.6
**IPPDq**	41.7	56.2	34.7	<LOQ	<LOQ	<LOQ
**DPPDq**	<LOQ	<LOQ	<LOQ	<LOQ	<LOQ	<LOQ
**6PPDq**	243	233	190	27.2	234	13.4
**CPPDq**	<LOQ	<LOQ	<LOQ	<LOQ	<LOQ	<LOQ
**DPPD**	2.33	3.94	3.05	<LOQ	<LOQ	<LOQ

**Hall 08 (ng/g)**	**FP A**	**FP B**	**FP C**	**SD A**	**SD B**	**SD C**
**Aniline**	294	394	204	312	100	54.7

**DPG**	28910	41110	28110	5431	2069	838
**2OH-BTZ**	22230	13890	18020	3432	5068	1775
**IPPD**	1577	4804	708	38.0	33.0	4.86
**BTZ**	99690	62080	57860	<LOQ	<LOQ	<LOQ
**2amino-BTZ**	1100	641	354	<LOQ	<LOQ	64.1
**2SH-BTZ**	203100	47480	59430	<LOQ	<LOQ	<LOQ
**HMMM**	234	865	1957	256	65.6	75.0
**CPPD**	13.1	7.00	14.3	<LOQ	<LOQ	<LOQ
**6PPD**	1332	6141	2869	296	20.4	25.0
**IPPDq**	109	70.2	25.4	7.20	28.8	<LOQ
**DPPDq**	<LOQ	<LOQ	<LOQ	<LOQ	<LOQ	<LOQ
**6PPDq**	179	232	140	78.6	<LOQ	6.34
**CPPDq**	<LOQ	<LOQ	<LOQ	<LOQ	<LOQ	<LOQ
**DPPD**	<LOQ	1.92	1.50	<LOQ	<LOQ	<LOQ

**Hall 09 (ng/g)**	**FP A**	**FP B**	**FP C**	**SD A**	**SD B**	**SD C**
**Aniline**	442	540	1111	172	165	264
**DPG**	32510	36590	67940	2861	1829	2445
**2OH-BTZ**	26810	30290	27920	1412	877	7454
**IPPD**	2330	2610	7454	35.2	22.6	20.2
**BTZ**	51760	60320	63930	<LOQ	<LOQ	26840
**2amino-BTZ**	628	841	1009	164	82.5	507
**2SH-BTZ**	76880	66250	119500	<LOQ	<LOQ	<LOQ
**HMMM**	24.1	24.4	23.8	41.0	31.0	67.3
**CPPD**	15.2	11.3	25.8	2.00	<LOQ	<LOQ
**6PPD**	2985	3113	7574	131	112	77.0
**IPPDq**	103	122	426	<LOQ	0.45	0.92
**DPPDq**	<LOQ	<LOQ	<LOQ	<LOQ	<LOQ	<LOQ
**6PPDq**	230	277	915	16.5	22.4	22.3
**CPPDq**	<LOQ	<LOQ	<LOQ	<LOQ	<LOQ	<LOQ
**DPPD**	6.58	7.21	13.0	<LOQ	<LOQ	<LOQ

**Shoe soles (ng/g)**	**SS 01**	**SS 02**	**SS 03**	**SS 04**	**SS 05**	**SS 06**	**SS 07**
**Aniline**	266	62.0	204	71.8	89.9	230	406
**DPG**	75.4	105	39.1	143	78.5	61.6	245
**2OH-BTZ**	66470	71960	38850	43730	60340	266900	32920
**IPPD**	100	22.4	7.40	23.2	8.60	50.7	10120
**BTZ**	77050	70820	54710	38980	42830	57070	50560
**2amino-BTZ**	2061	2563	1267	1439	1043	607	668
**2SH-BTZ**	407800	489300	576500	556300	437400	1431000	379000
**HMMM**	24.0	57.8	7.10	38.9	7.70	46.4	3.00
**CPPD**	<LOQ	<LOQ	<LOQ	<LOQ	<LOQ	<LOQ	210
**6PPD**	40.0	4.20	<LOQ	5.80	<LOQ	4.80	11710

**IPPDq**	13.9	9.10	7.60	9.90	6.60	2.10	343
**DPPDq**	<LOQ	<LOQ	<LOQ	<LOQ	<LOQ	34.90	<LOQ
**6PPDq**	22.2	13.3	9.90	19.4	8.80	1.10	371
**CPPDq**	<LOQ	<LOQ	<LOQ	<LOQ	<LOQ	2.80	3.90
**DPPD**	<LOQ	<LOQ	<LOQ	<LOQ	<LOQ	18.4	18.7
	**SS 08**	**SS 09**	**SS 10**	**SS 11**	**SS 12**	**SS 13**	**SS 14**
**Aniline**	<LOQ	<LOQ	1653	<LOQ	965	<LOQ	2050
**DPG**	<LOQ	<LOQ	2061	22.9	<LOQ	170	<LOQ
**2OH-BTZ**	10450	7366	9954	9719	5540	101500	8478
**IPPD**	12.6	11.3	11.8	12.5	13.3	719	16.3
**BTZ**	23150	20050	44410	20670	12350	28580	43240
**2amino-BTZ**	16470	<LOQ	<LOQ	2988	<LOQ	19200	<LOQ
**2SH-BTZ**	155700	64160	470900	198600	233500	116000	142600
**HMMM**	<LOQ	78.7	<LOQ	<LOQ	<LOQ	<LOQ	<LOQ
**CPPD**	<LOQ	<LOQ	<LOQ	<LOQ	<LOQ	<LOQ	<LOQ
**6PPD**	361	<LOQ	<LOQ	207	183	464	278
**IPPDq**	<LOQ	<LOQ	<LOQ	<LOQ	<LOQ	<LOQ	<LOQ
**DPPDq**	<LOQ	<LOQ	<LOQ	<LOQ	<LOQ	<LOQ	<LOQ
**6PPDq**	<LOQ	<LOQ	<LOQ	<LOQ	<LOQ	<LOQ	<LOQ
**CPPDq**	<LOQ	<LOQ	<LOQ	<LOQ	<LOQ	<LOQ	<LOQ
**DPPD**	<LOQ	<LOQ	129	98.3	<LOQ	<LOQ	<LOQ
	**SS 15**	**SS 16**	**SS 17**	**SS 18**	**SS 19**	**SS 20**	**SS 21**
**Aniline**	<LOQ	87.4	225	16460	224700	840	237
**DPG**	<LOQ	223	5.60	625000	813600	9289	13600
**2OH-BTZ**	14280	8957	90520	79140	77000	42270	12430
**IPPD**	8301	<LOQ	5.90	<LOQ	<LOQ	18.9	5.80
**BTZ**	78550	8137	61610	100200	228100	201700	56920
**2amino-BTZ**	<LOQ	17.7	109	369	647	<LOQ	<LOQ
**2SH-BTZ**	138600	5889	8173	19150	2061000	2366000	719000
**HMMM**	<LOQ	15.0	<LOQ	0.10	3.10	2.80	1.00
**CPPD**	180	<LOQ	<LOQ	132	8.80	156	<LOQ
**6PPD**	22600	1123	2.20	1.50	1.30	1.40	0.40
**IPPDq**	54.2	<LOQ	<LOQ	<LOQ	<LOQ	<LOQ	<LOQ
**DPPDq**	<LOQ	<LOQ	<LOQ	<LOQ	<LOQ	<LOQ	<LOQ
**6PPDq**	83.6	89.1	10.7	1.30	1.00	1.10	12.8
**CPPDq**	<LOQ	<LOQ	<LOQ	6.70	<LOQ	<LOQ	<LOQ
**DPPD**	<LOQ	<LOQ	<LOQ	<LOQ	3.3	19.4	2.10
	**SS 22**	**SS 23**	**SS 24**	**SS 25**	**SS 26**	**SS 27**	**SS 28**
**Aniline**	219	1214	183	271	262	1903	32.5
**DPG**	6796	1511	73	2169	423	364	58.3
**2OH-BTZ**	17190	33590	19380	13540	372600	33500	15330
**IPPD**	2.50	1.20	2.30	5.20	17.6	202	0.90
**BTZ**	60120	37410	6924	52370	5108	112400	28130

**2amino-BTZ**	28	378	45	<LOQ	534	1031	130
**2SH-BTZ**	483300	1472000	804500	580700	415800	1342000	24510
**HMMM**	1.20	1.20	3.10	1.70	0.70	2.60	0.80
**CPPD**	<LOQ	<LOQ	<LOQ	<LOQ	<LOQ	<LOQ	<LOQ
**6PPD**	0.30	11	301	614	133	896	2.90
**IPPDq**	<LOQ	<LOQ	<LOQ	<LOQ	<LOQ	<LOQ	<LOQ
**DPPDq**	<LOQ	<LOQ	<LOQ	<LOQ	<LOQ	<LOQ	<LOQ
**6PPDq**	9.90	9.50	2.40	4.80	6.40	15.9	1.40
**CPPDq**	<LOQ	<LOQ	<LOQ	<LOQ	<LOQ	<LOQ	<LOQ
**DPPD**	<LOQ	<LOQ	14.1	<LOQ	<LOQ	54.9	<LOQ

	**SS 29**	**SS 30**
**Aniline**	555	<LOQ
**DPG**	117	12.0
**2OH-BTZ**	8520	3281
**IPPD**	148	7.39
**BTZ**	8482	14300
**2amino-BTZ**	16550	<LOQ
**2SH-BTZ**	5392	46580
**HMMM**	90.2	429
**CPPD**	<LOQ	<LOQ
**6PPD**	46.2	<LOQ
**IPPDq**	15.4	<LOQ
**DPPDq**	<LOQ	<LOQ
**6PPDq**	<LOQ	<LOQ
**CPPDq**	<LOQ	<LOQ
**DPPD**	<LOQ	<LOQ

Figure S1: Literature comparison of RDCs in aerosol particulate matter and settled dust samples, which based on our data, do not necessarily arise uniquely from climbing activity. Compounds in light gray were either below limit of quantification or very sporadically detected at trace concentrations. Details about literature values provided in the Supporting Information: Excel file. Statistical differences between groups were tested with the Wilcoxon signed-rank test (NS means $p \geq 0.05$; * means $p < 0.05$; ** means $p < 0.01$; *** means $p < 0.001$).

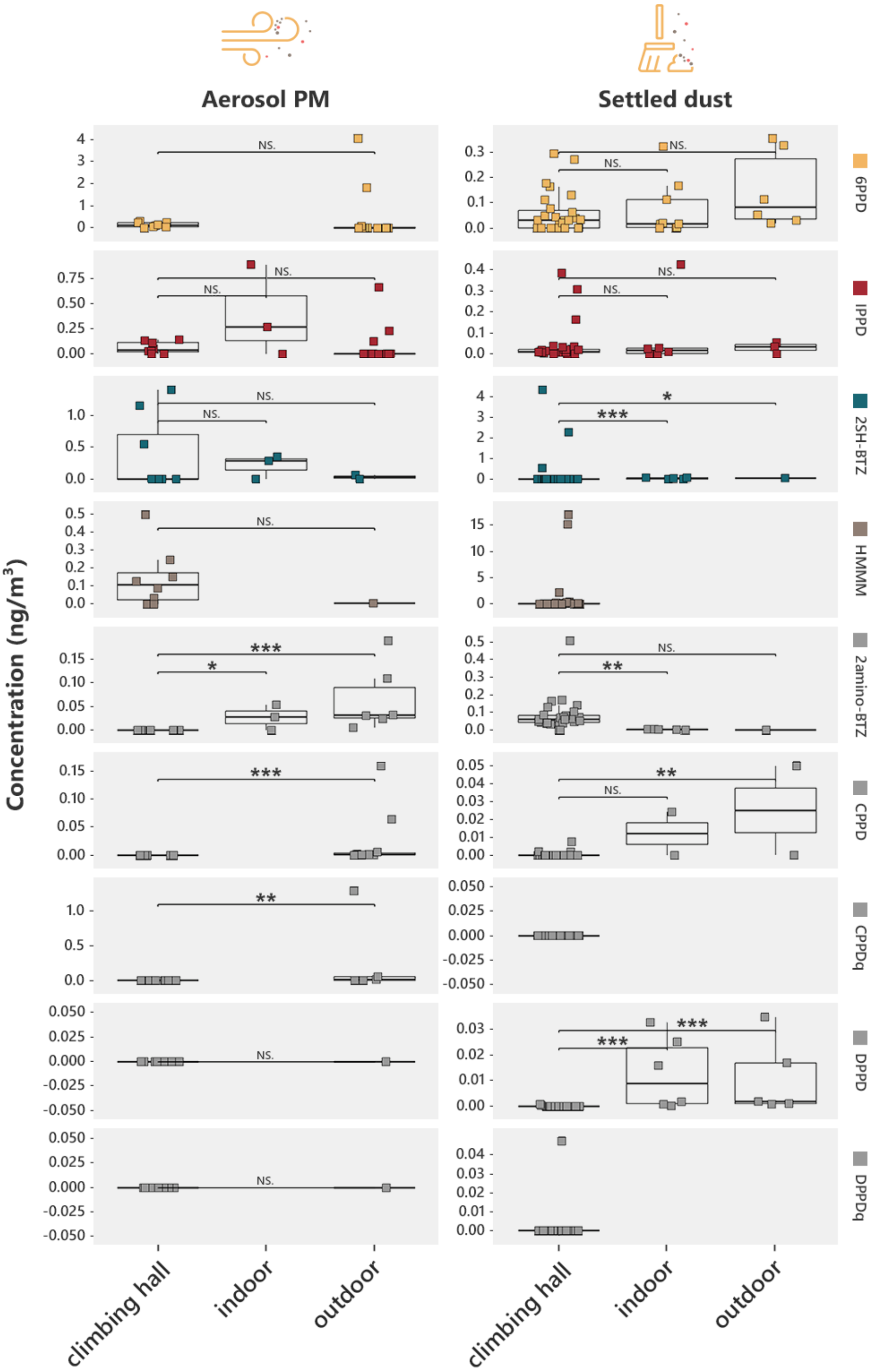

Figure S2: RDC profile in all sample replicates from each hall

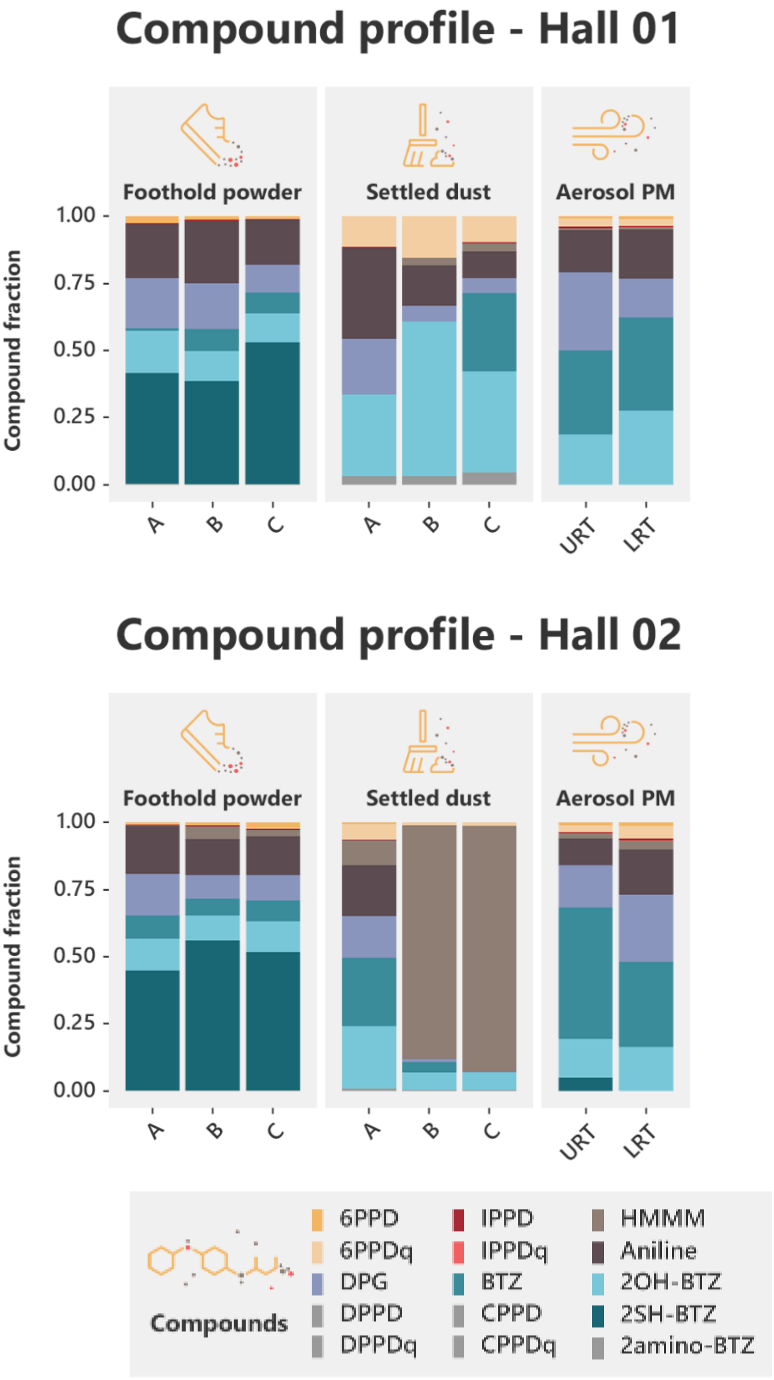

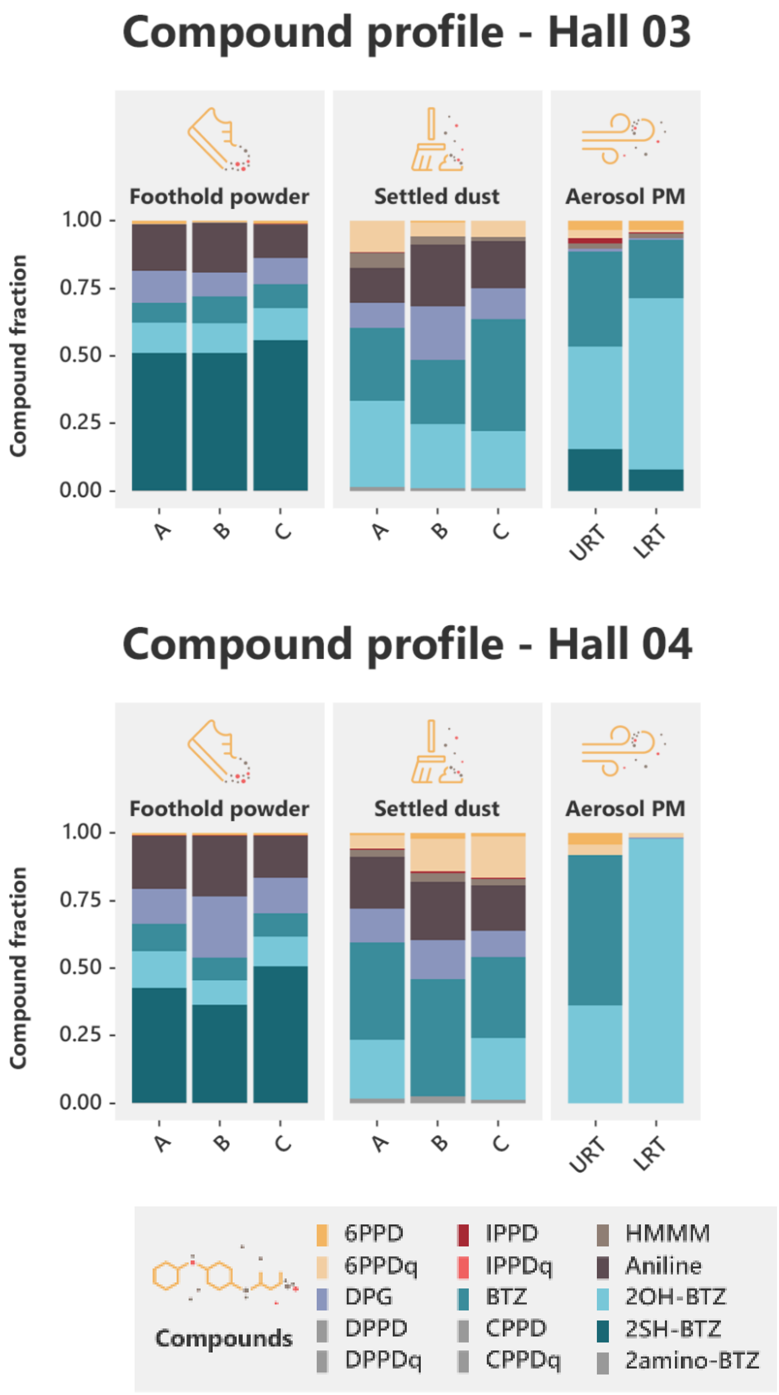

Compound profile - Hall 03
Foothold powder
Settled dust
Aerosol PM
Compound fraction
1.00
0.75
0.50
0.25
0.00
A
B
C
A
B
C
URT
LRT
Compound profile - Hall 04
Foothold powder
Settled dust
Aerosol PM
Compound fraction
1.00
0.75
0.50
0.25
0.00
A
B
C
A
B
C
URT
LRT
Compounds
6PPD
6PPDq
DPG
DPPD
DPPDq
IPPD
IPPDq
BTZ
CPPD
CPPDq
HMMM
Aniline
2OH-BTZ
2SH-BTZ
2amino-BTZ

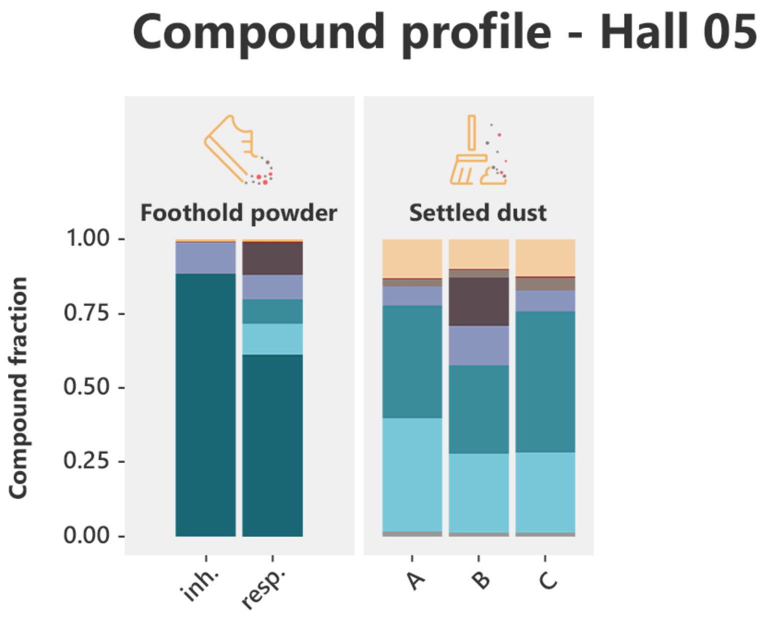
Compound profile - Hall 05
Foothold powder
Settled dust
Compound fraction
1.00
0.75
0.50
0.25
0.00
inh.
resp.
A
B
C

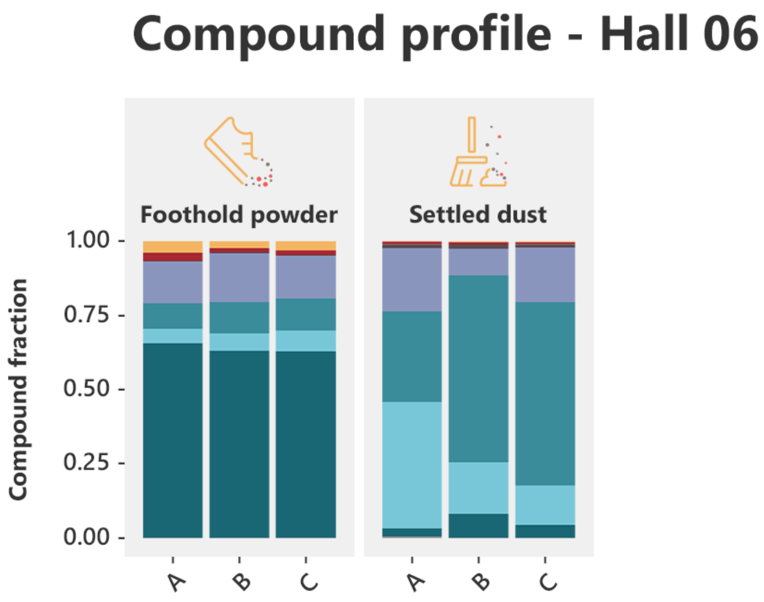
Compound profile - Hall 06
Foothold powder
Settled dust
Compound fraction
1.00
0.75
0.50
0.25
0.00
A
B
C
A
B
C

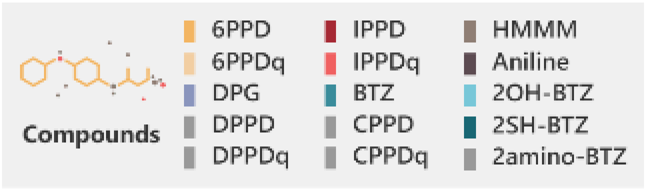
Compounds
6PPD
6PPDq
DPG
DPPD
DPPDq
IPPD
IPPDq
BTZ
CPPD
CPPDq
HMMM
Aniline
2OH-BTZ
2SH-BTZ
2amino-BTZ

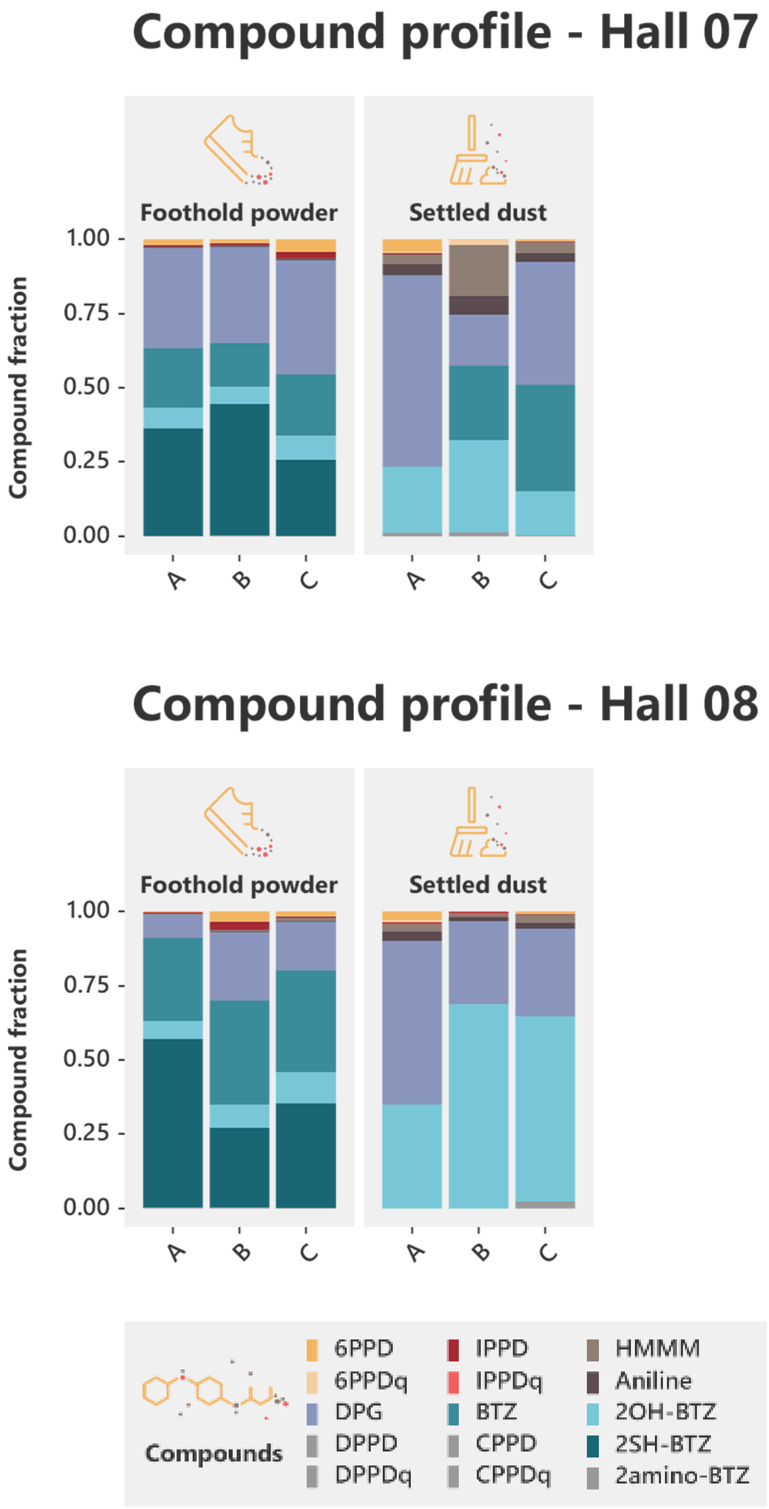
Compound profile - Hall 07
Foothold powder
Settled dust
Compound fraction
1.00
0.75
0.50
0.25
0.00
A
B
C
Compound profile - Hall 08
Foothold powder
Settled dust
Compound fraction
1.00
0.75
0.50
0.25
0.00
A
B
C
Compounds
6PPD
6PPDq
DPG
DPPD
DPPDq
IPPD
IPPDq
BTZ
CPPD
CPPDq
HMMM
Aniline
2OH-BTZ
2SH-BTZ
2amino-BTZ

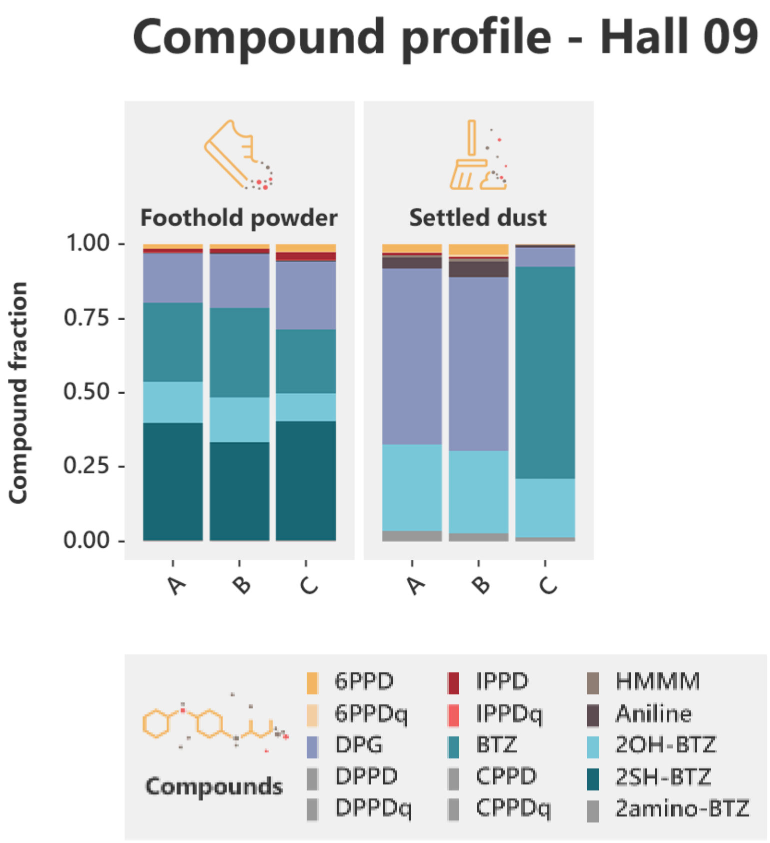
Compound profile - Hall 09
Foothold powder
Settled dust
Compound fraction
1.00
0.75
0.50
0.25
0.00
A
B
C
A
B
C
Compounds
6PPD
6PPDq
DPG
DPPD
DPPDq
IPPD
IPPDq
BTZ
CPPD
CPPDq
HMMM
Aniline
2OH-BTZ
2SH-BTZ
2amino-BTZ

Figure S3: Rubber-derived compound profile in shoe soles and foothold powder

Profile showing $[\text{compound}_i]/\sum 15[\text{compounds}]$ for 15 rubber-derived compounds in 30 shoe sole samples, and foothold powder from nine climbing halls. The rubber-derived compound profile varies between shoe sole models, reflecting the variety of targeted properties in different climbing shoes (adhesiveness, softness, durability, flexibility). The foothold powder samples have a rubber-derived compound profile which represents an average of the different shoes on the market, and is relatively consistent between halls.

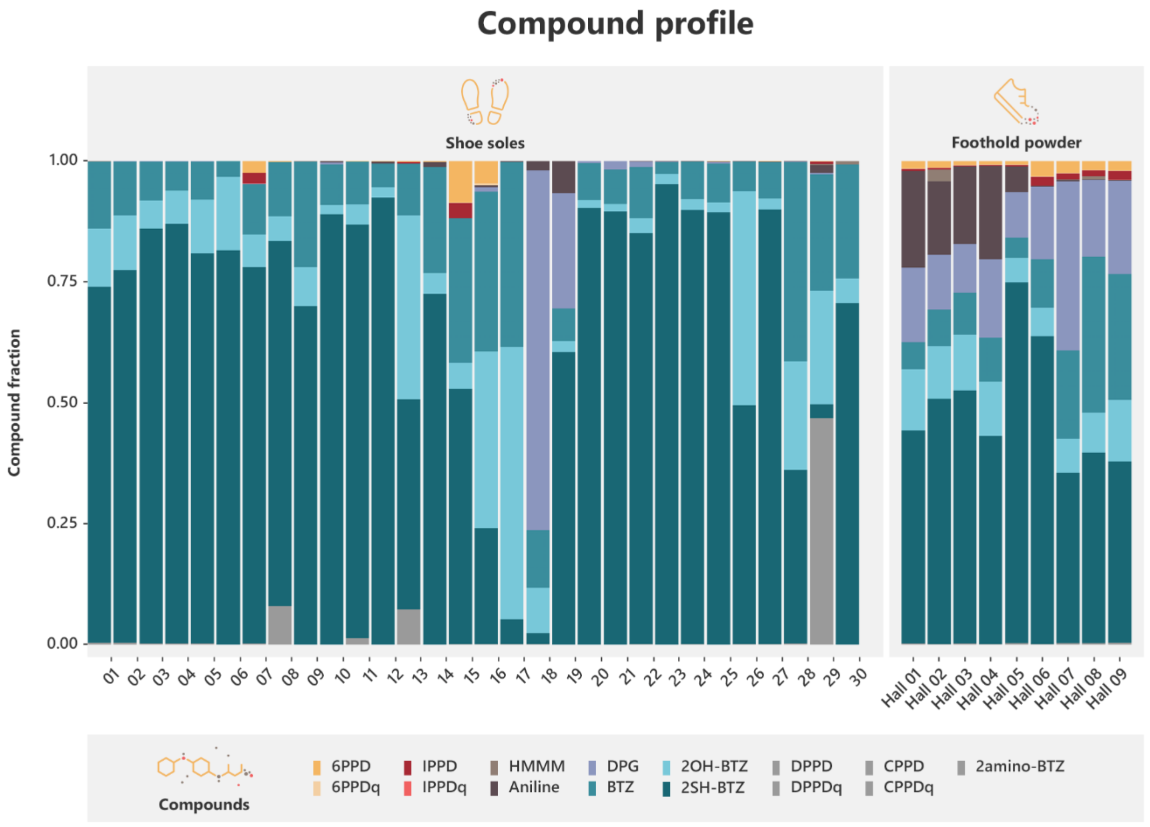

Figure S4: Rubber-derived compound concentration shifts.
Concentration % (calculated as $[compound_i]/\sum_{15}[compounds]\times100$) for 2-mercaptobenzothiazole, 2-hydroxybenzothiazole, benzothiazole, 6PPD, 6PPD-quinone, IPPD, and IPPD-quinone in foothold powder (FP), settled dust (SD), and aerosol particulate matter (APM) samples. Compounds shown in blue are most likely included in climbing shoe formulation as additives, while compounds shown in red are most likely transformation products of those additives. Relative concentrations of 2-mercaptobenzothiazole, 6PPD, and IPPD decrease from foothold powder to settled dust and aerosol particulate matter samples, likely due to transformation. Meanwhile, relative concentrations of products 2-hydroxybenzothiazole, benzothiazole, 6PPD-quinone, and IPPD-quinone increase.

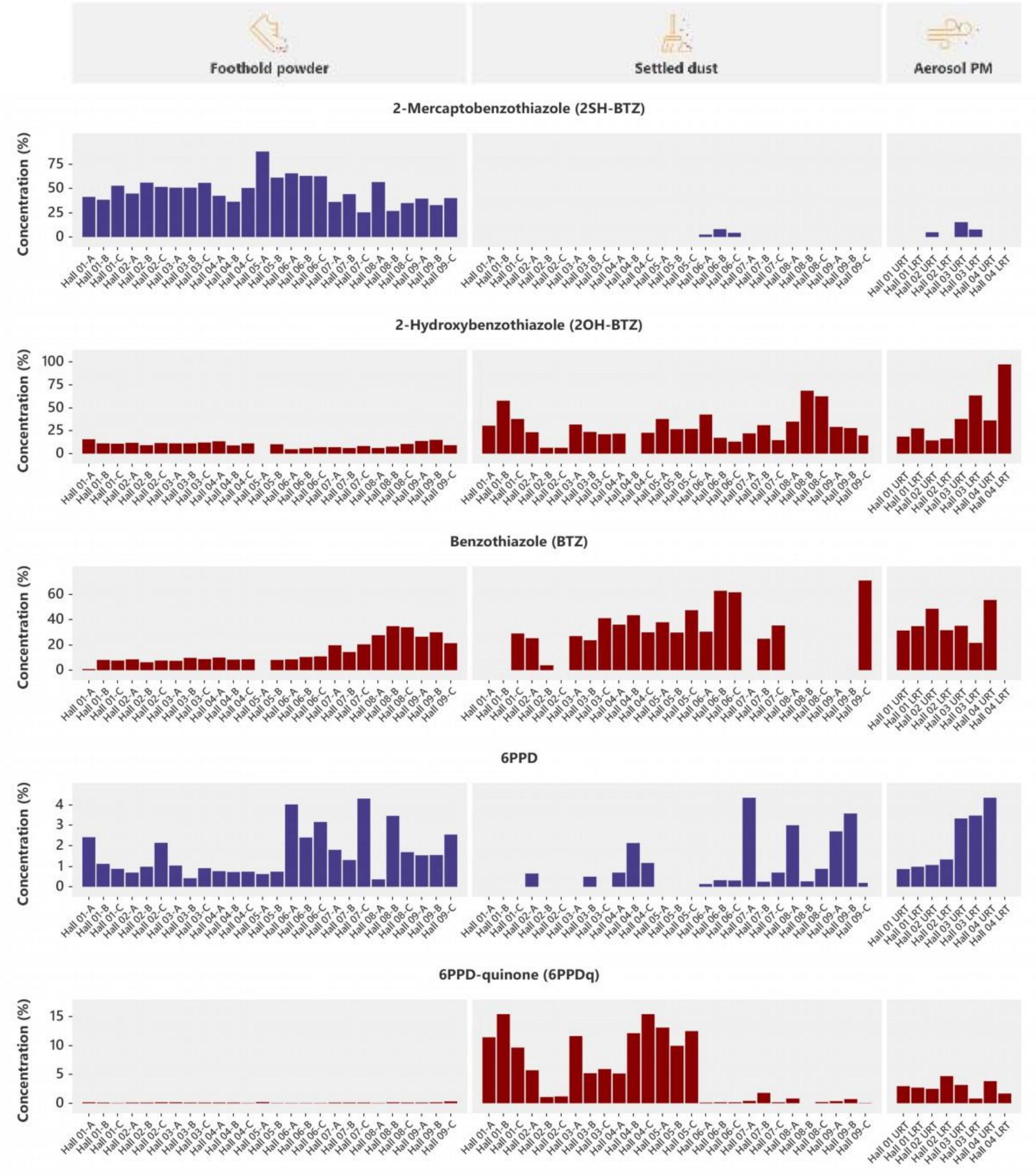

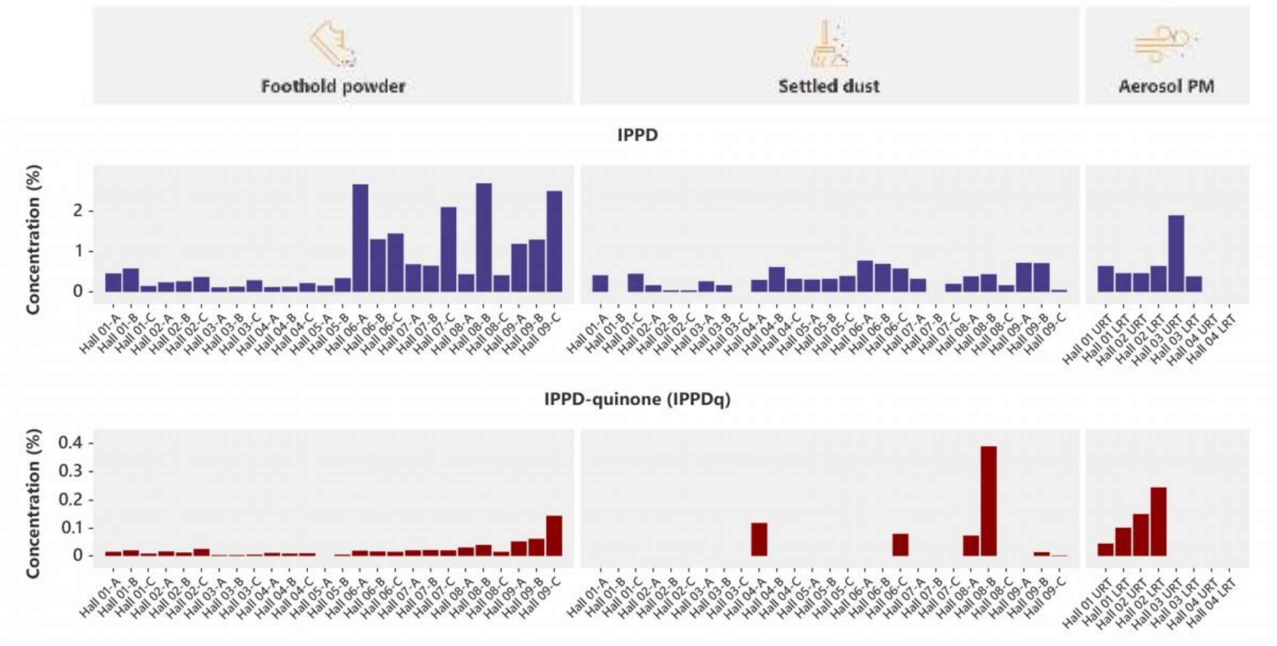
Foothold powder
Settled dust
Aerosol PM
IPPD
Concentration (%)
0
1
2
IPPD-quinone (IPPDq)
Concentration (%)
0
0.1
0.2
0.3
0.4
Hall 01-A
Hall 01-B
Hall 01-C
Hall 02-A
Hall 02-B
Hall 02-C
Hall 03-A
Hall 03-B
Hall 03-C
Hall 04-A
Hall 04-B
Hall 04-C
Hall 05-A
Hall 05-B
Hall 05-C
Hall 06-A
Hall 06-B
Hall 06-C
Hall 07-A
Hall 07-B
Hall 07-C
Hall 08-A
Hall 08-B
Hall 08-C
Hall 09-A
Hall 09-B
Hall 09-C
Hall 01 URT
Hall 01 LRT
Hall 02 URT
Hall 02 LRT
Hall 03 URT
Hall 03 LRT
Hall 04 URT
Hall 04 LRT

Table S5: Ozonation Experimental Results and p-values representing the statistically significance of a difference in concentrations before and after ozonation. P-values for absolute concentrations (shown in this table), and for relative concentrations (normalized to ΣRDCs) were computed using a Student's t-test.

	**No ozonation (µg/g)**			**4 hours ozonation (µg/g)**			**p-value absolute concentration**	**p-value relative concentration**
**Aniline**	34.5	29.5	30.9	12.1	12.1	8.9	0.0006	0.009
**DPG**	36.9	29.4	29.1	12.4	11.6	8.4	0.006	0.0003
**2OH-BTZ**	16.7	15.7	13.3	21.7	18.9	10.2	0.7	0.04
**IPPD**	5.4	2.5	2.1	2.3	1.2	1.1	0.2	0.4
**BTZ**	13.4	11.0	15.0	44.4	31.7	41.5	0.01	0.03
**2amino-BTZ**	0.2	0.2	<LOQ	0.2	0.2	<LOQ	0.6	0.3
**2SH-BTZ**	101.2	68.3	64.2	30.9	26.7	11.3	0.03	0.02
**HMMM**	0.05	0.05	0.06	0.04	0.04	0.02	0.06	1
**CPPD**	0.1	<LOQ	<LOQ	<LOQ	<LOQ	<LOQ	NA	NA
**6PPD**	7.9	2.5	2.4	1.5	0.8	0.6	0.2	0.2
**IPPDq**	0.08	0.06	0.05	0.06	0.04	0.05	0.4	0.1
**DPPDq**	<LOQ	<LOQ	<LOQ	<LOQ	<LOQ	<LOQ	NA	NA
**6PPDq**	0.2	0.1	0.1	0.1	0.1	0.1	0.08	0.01
**CPPDq**	<LOQ	<LOQ	<LOQ	<LOQ	<LOQ	<LOQ	NA	NA
**DPPD**	<LOQ	<LOQ	<LOQ	<LOQ	<LOQ	<LOQ	NA	NA

Figure S5: Ozonation experiment results

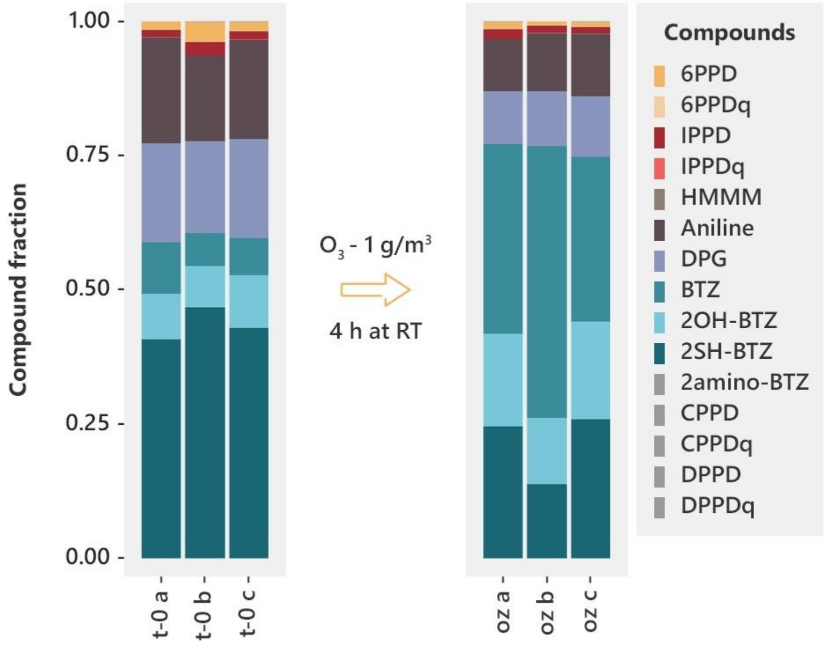

Figure S6: Rubber derived compound concentration shifts ozonation experiments

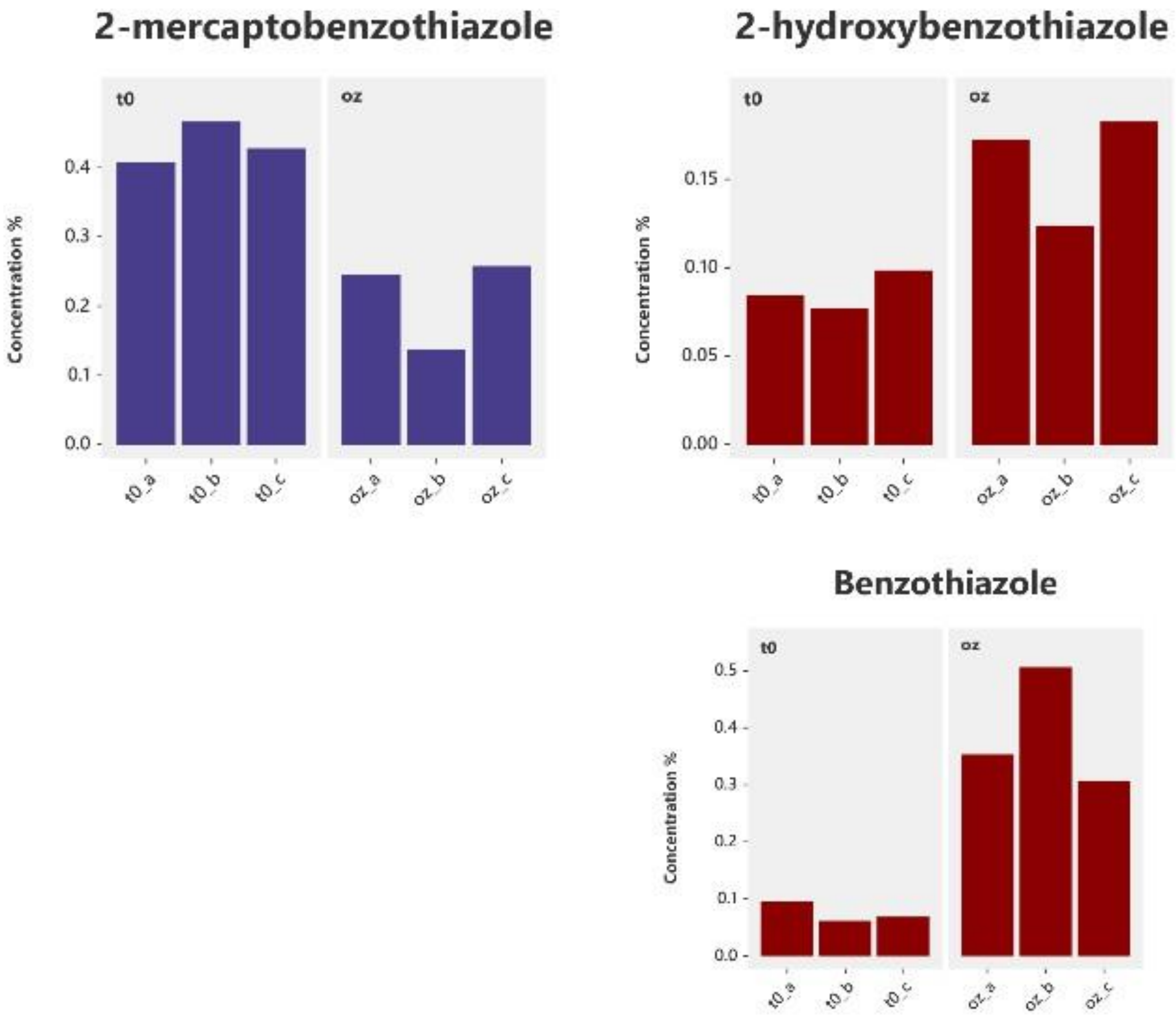

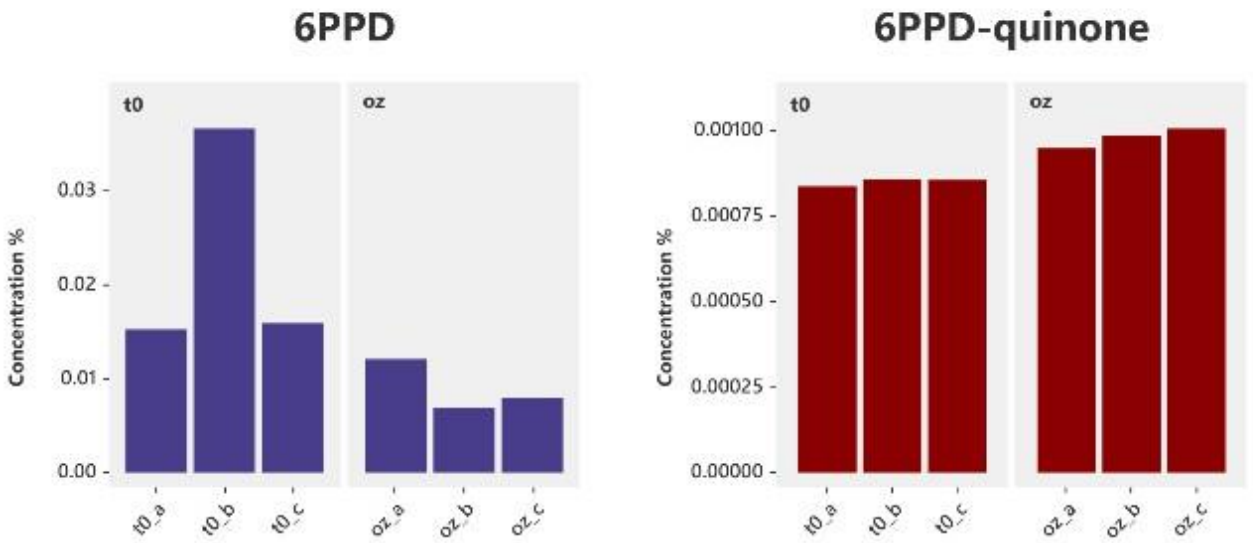
6PPD
6PPD-quinone
t0
oz
Concentration %
0.00
0.01
0.02
0.03
0.00000
0.00025
0.00050
0.00075
0.00100
t0_a
t0_b
t0_c
oz_a
oz_b
oz_c

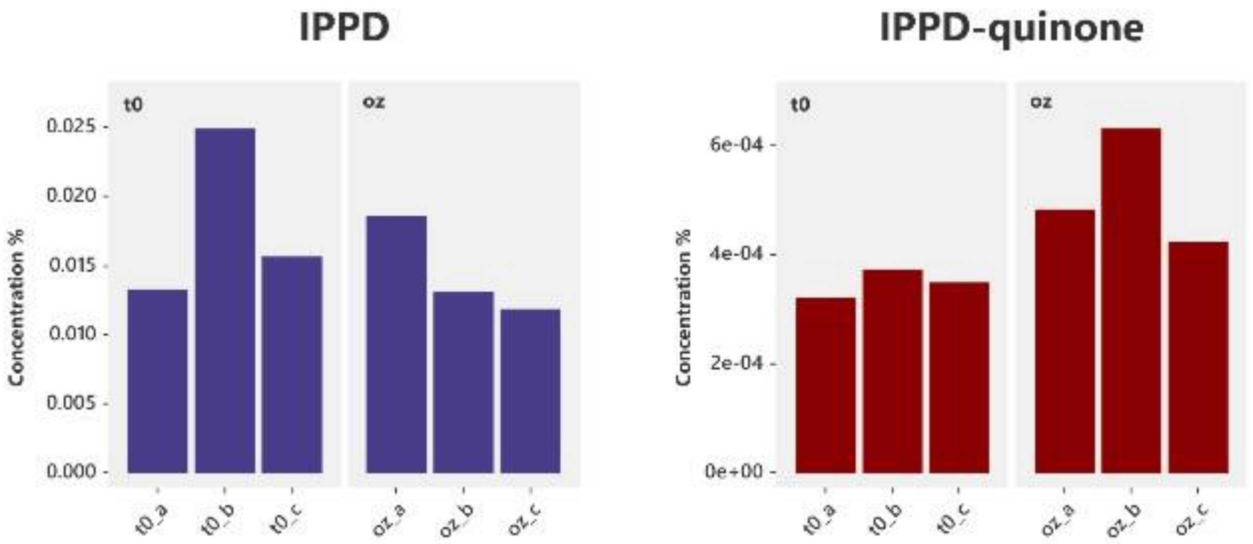
IPPD
IPPD-quinone
t0
oz
Concentration %
0.000
0.005
0.010
0.015
0.020
0.025
0e+00
2e-04
4e-04
6e-04
t0_a
t0_b
t0_c
oz_a
oz_b
oz_c

Figure S7: EDX imaging of a foothold powder particle.

The elongated shape results from the abrasion process, and is commonly observed for tire wear particles. Elemental analysis shows the abondance of C, O and Si representing the carbon backbone of the rubber matrix, excluding the probability of inorganic particles such as chalk particles. Mg is also present and could come from the rubber matrix or from chalk particles (MgCO3) that deposited on the surface.

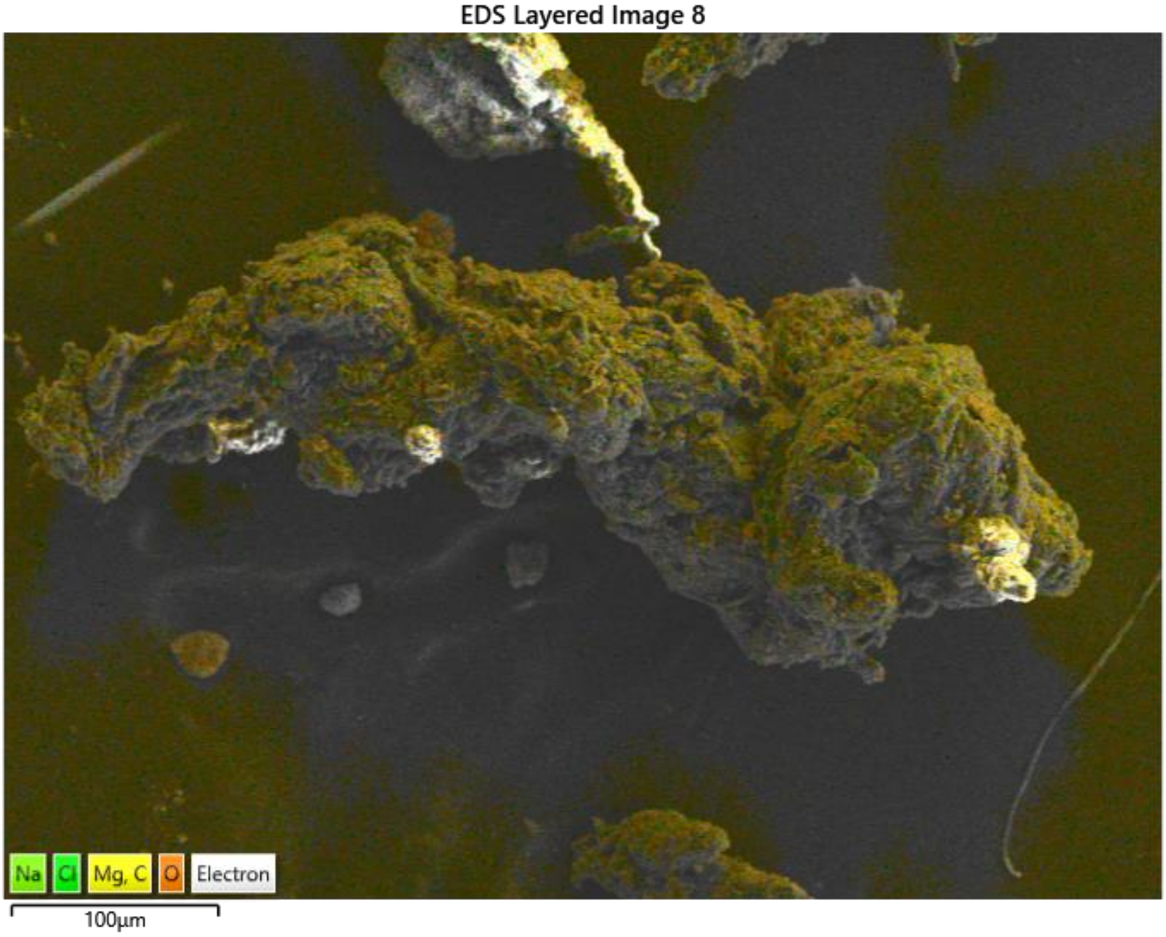

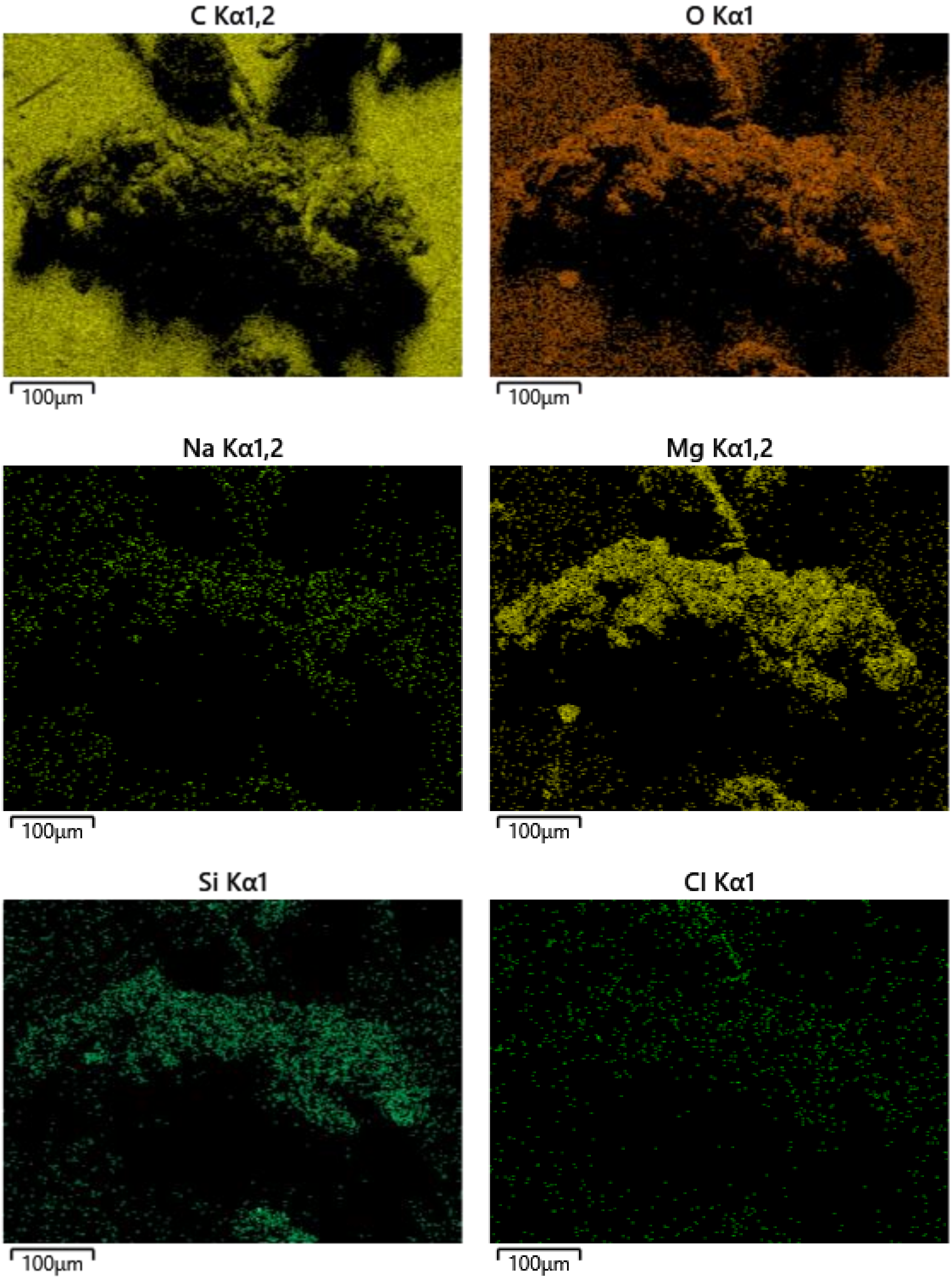
C Kα1,2
100µm
O Kα1
100µm
Na Kα1,2
100µm
Mg Kα1,2
100µm
Si Kα1
100µm
Cl Kα1
100µm

Table S6: Physico-chemical characteristics of Rubber derived chemicals

Compound	CAS	Log K_{OA}	Modelled % on particle phase at equilibrium	Measured particle-bound fraction (%) in ambient $PM_{2.5}$[14]
Aniline	62-53-3	5.3[15]	/	/
BTZ	95-16-9	6.0[16]- 6.8[17]	0.0[17]	25
2amino-BTZ	136-95-8	10.3[17]	10.4[17]	55
2OH-BTZ	934-34-9	8.4[17]	0.1[17]	5
2SH-BTZ	149-30-4	11.0[17]	48.6[17]	/
6PPDq	2754428-18-5	15.3[18]	35 – 100[18]	98
DPG	102-06-7	12.4[17]	94.3[17]	95
6PPD	793-24-8	9.3[17]	1.2[17]	100
HMMM	3089-11-0	/	/	75
CPPD	101-87-1	9.3[17]	1.4[17]	/
CPPDq	68054-78-4	/	/	/
IPPD	101-72-4	10.5[17]	17.7[17]	100
IPPDq	68054-73-9	/	/	75
DPPD	74-31-7	12.5[19] - 12.1[17]	87.8[17]	/
DPPDq	3421-08-7	/	/	/

References

1. Organization, W. H. WHO Global Air Quality Guidelines: Particulate Matter (PM2.5 and PM10), Ozone, Nitrogen Dioxide, Sulfur Dioxide and Carbon Monoxide. (World Health Organization, Geneva PP - Geneva).
2. Harrison, R. M., Giorio, C., Beddows, D. C. S. & Dall'Osto, M. Size distribution of airborne particles controls outcome of epidemiological studies. Science of The Total Environment **409**, 289–293 (2010).
3. Ferro, A. & Hidemann, L. Inhalation Exposure, Uptake, and Dose. in Exposure Analysis (eds. Ott, W. R., Steinemann, A. C. & Wallace, L. A.) (CRC Press, New York, 2006).
4. Schreder, E. D., Uding, N. & La Guardia, M. J. Inhalation a significant exposure route for chlorinated organophosphate flame retardants. Chemosphere **150**, 499–504 (2016).
5. World Health Organization - Occupational and Environmental Health Team. Hazard prevention and control in the work environment: : airborne dust. At head of title: Prevention and Control Exchange Preprint at (1999).
6. Scheuch, G., Kohlhaeufl, M. J., Brand, P. & Siekmeier, R. Clinical perspectives on pulmonary systemic and macromolecular delivery. Advanced Drug Delivery Reviews **58**, 996–1008 (2006).
7. Janssen, N. A. H., Fischer, P., Marra, M., Ameling, C. & Cassee, F. R. Short-term effects of PM2.5, PM10 and PM2.5–10 on daily mortality in the Netherlands. Science of The Total Environment **463–464**, 20–26 (2013).
8. Chen, J. & Hoek, G. Long-term exposure to PM and all-cause and cause-specific mortality: A systematic review and meta-analysis. Environment International **143**, 105974 (2020).
9. Orellano, P., Reynoso, J., Quaranta, N., Bardach, A. & Ciapponi, A. Short-term exposure to particulate matter (PM10 and PM2.5), nitrogen dioxide (NO2), and ozone (O3) and all-cause and cause-specific mortality: Systematic review and meta-analysis. Environment International **142**, 105876 (2020).
10. Hallworth, G. W. & Westmoreland, D. G. The twin impinger: a simple device for assessing the delivery of drugs from metered dose pressurized aerosol inhalers. Journal of Pharmacy and Pharmacology **39**, 966–972 (2011).

11. Yu, K.-P., Chen, Y.-P., Gong, J.-Y., Chen, Y.-C. & Cheng, C.-C. Improving the collection efficiency of the liquid impinger for ultrafine particles and viral aerosols by applying granular bed filtration. Journal of Aerosol Science **101**, 133–143 (2016).

12. Lin, X., Willeke, K., Ulevicius, V. & Grinshpun, S. A. Effect of Sampling Time on the Collection Efficiency of All-Glass Impingers. American Industrial Hygiene Association Journal **58**, 480–488 (1997).

13. US Environmental Protection Agency. Exposure Factors Handbook: 2011 Edition. U.S. Environmental Protection Agency **EPA/600/R-**, 1–1466 (2011).

14. Tian, L. et al. Tire Wear Chemicals in the Urban Atmosphere: Significant Contributions of Tire Wear Particles to $PM_{2.5}$. Environ. Sci. Technol. acs.est.4c04378 (2024) doi:10.1021/acs.est.4c04378.

15. Abraham, M. H., Acree, Jr., W. E., Leo, A. J. & Hoekman, D. Partition of compounds from water and from air into the wet and dry monohalobenzenes. New J. Chem. **33**, 1685 (2009).

16. Ariyasena, T. C. & Poole, C. F. Determination of descriptors for polycyclic aromatic hydrocarbons and related compounds by chromatographic methods and liquid–liquid partition in totally organic biphasic systems. Journal of Chromatography A **1361**, 240–254 (2014).

17. Johannessen, C., Liggio, J., Zhang, X., Saini, A. & Harner, T. Composition and transformation chemistry of tire-wear derived organic chemicals and implications for air pollution. Atmospheric Pollution Research **13**, 101533 (2022).

18. Environmental Assessment Program and Water Quality Program. 6PPD in Road Runoff: Assessment and Mitigation Strategies. https://apps.ecology.wa.gov/publications/summarypages/2203020.html (2022).

19. Karunasekara, T. & Poole, C. F. Models for liquid–liquid partition in the system dimethyl sulfoxide–organic solvent and their use for estimating descriptors for organic compounds. Journal of Chromatography A (2011) doi:10.1016/j.chroma.2011.05.023.
